# Emerging Chimeric Antigen Receptor-Immune Cell Therapy for Pancreatic Cancer: Mechanisms, Clinical Advances, and Future Perspectives

**DOI:** 10.32604/or.2025.073554

**Published:** 2026-03-23

**Authors:** Shuo Wang, Miao Wang, Wei Yao

**Affiliations:** Department of General Surgery, Shengjing Hospital of China Medical University, No. 36, San Hao Street, Heping District, Shenyang, 110004, China

**Keywords:** Chimeric antigen receptor, pancreatic cancer, immune cell therapy, immunotherapy, clinical trial

## Abstract

Pancreatic ductal adenocarcinoma (PDAC) remains one of the most lethal malignancies, characterized by a highly immunosuppressive tumor microenvironment (TME), dense stromal architecture, and limited response to conventional therapies. This review comprehensively examines the emerging role of chimeric antigen receptor (CAR)-engineered immune cells, including chimeric antigen receptor-T (CAR-T), CAR-macrophages (CAR-M), and CAR-natural killer (CAR-NK) cells, as innovative immunotherapeutic strategies for PDAC. We delve into the mechanistic foundations of these platforms, highlighting their unique abilities to target tumor-associated antigens, overcome stromal barriers, and remodel the immunosuppressive TME. Recent preclinical and clinical advances demonstrate promising antitumor activity, particularly with targets such as mesothelin, claudin18.2, and human epidermal growth factor 2 (HER2), though challenges related to antigen heterogeneity, TME suppression, and cell persistence remain. We further discuss synergistic approaches involving genetic engineering, microenvironment modulation, and combination therapies aimed at enhancing efficacy. Finally, we offer perspectives on the future direction of CAR-based therapies, including the development of next-generation constructs, allogeneic “off-the-shelf” products, and personalized combination regimens, underscoring their potential in pancreatic cancer.

## 1 Introduction

### 1.1 The Global Burden of Pancreatic Ductal Adenocarcinoma

Pancreatic ductal adenocarcinoma (PDAC) continues to pose a persistent and heavy burden in human health, distinguished by its insidious onset, aggressively biological behavior, and notoriously dismal prognosis [1]. As the fourth most common cause of cancer-related mortality in the United States and the sixth worldwide, PDAC was responsible for an estimated 510,922 new diagnoses and 467,409 deaths globally in 2022 alone. Strikingly, projections suggest that pancreatic cancer is poised to become the second leading cause of cancer-related deaths in the U.S. by 2030 [2]. Further underscoring its growing burden, current models indicate a staggering 95.4% rise in global incidence by 2050, potentially approaching one million new cases annually [3]. The five-year survival rate remains dismally low at approximately 6%–10%, the poorest among all major solid tumors and virtually unchanged over the past forty years [4]. This dire outlook stands in stark discrepancy with the substantial advances achieved across other cancer types, where aggregate five-year survival rates now exceed 67% for all malignancies combined [5]. Compounding these challenges, the clinical management of PDAC is greatly hindered by its asymptomatic early progression, with 80%–85% of patients presenting with locally advanced or metastatic disease at diagnosis, thereby rendering the majority ineligible for curative surgical resection [6,7].

### 1.2 Limitations of Current Therapies of PDAC

The treatment paradigm for pancreatic cancer continues to be largely defined by surgical resection and chemotherapy, albeit with persistently limited success [8]. Surgical intervention, particularly the Whipple procedure (pancreaticoduodenectomy), remains the only potentially curative option; however, only 15%–20% of patients present with resectable disease at diagnosis [9]. Even among those eligible for surgery, recurrence rates remain unacceptably high [10]. Systemic chemotherapy, mainly involving gemcitabine-based protocols or FOLFIRINOX (a combination of oxaliplatin, irinotecan, fluorouracil, and leucovorin), confers only modest survival benefits, typically extending life by months rather than years [11]. Although the modified FOLFIRINOX regimen demonstrates superior efficacy compared to gemcitabine monotherapy, median survival remains under one year, and treatment is frequently accompanied by substantial toxicity [12]. Radiotherapy, including both conventional photon-based techniques and advanced modalities such as carbon-ion radiotherapy, has achieved limited success, hampered by intrinsic radioresistance and anatomical constraints that prevent delivery of tumoricidal doses without risking damage to adjacent organs [13]. Moreover, emerging evidence indicates that standard chemoradiation may inadvertently induce epithelial-mesenchymal transition (EMT) in residual tumor cells, potentially enhancing their invasive behavior and fostering therapeutic resistance [14].

### 1.3 What Makes the PDAC Microenvironment so Resistant?

The emergence of targeted therapies and immunotherapies initially inspired considerable optimism, which has since been moderated by an improved understanding of PDAC’s complex and hostile tumor biology. The tumor microenvironment (TME) of pancreatic cancer is notably immunosuppressive, dominated by a dense desmoplastic stroma that can comprise up to 90% of the tumor mass. This fibrous network acts as a physical barrier, severely impeding immune cell infiltration [15,16]. This stroma is populated by cancer-associated fibroblasts (CAFs), regulatory T cells (Tregs), myeloid-derived suppressor cells (MDSCs), and M2 macrophages, which collaboratively sustain an immunologically cold microenvironment [17,18]. Furthermore, pancreatic tumors generally display a low tumor mutational burden and impaired antigen presentation machinery, which together diminish T-cell activation and effector responses [19]. As a result, single-agent immunotherapies—including immune checkpoint inhibitors (ICIs) that have transformed outcomes in many other cancers—have shown limited clinical activity in PDAC, except in the uncommon subset of tumors with mismatch repair deficiency [20,21].

### 1.4 A New Frontier: Harnessing Immune Cells to Combat PDAC

Within this constrained therapeutic landscape, immune cell-based therapies have arisen as a promising new strategic direction [22]. The scientific rationale for this approach lies in its potential to overcome the core limitations of conventional modalities by leveraging and augmenting the innate capacity of immune cells to identify and eradicate malignant cells. Recent clinical investigations have begun to substantiate this promise. For example, latest trial evaluating CT041, a chimeric antigen receptor (CAR)-T therapy targeting claudin18.2, enrolled 24 pancreatic cancer patients and observed objective tumor reduction in 12 individuals. The disease control rate (DCR) reached 70.8%, with a median overall survival (mOS) of 10.0 months and a one-year survival rate of 45.8%. Additionally, the median duration of response (DOR) was 9.5 months, with a 50% response persistence rate at one year [23]. For patients with advanced PDAC, including both locally advanced and metastatic disease, current standard multiagent cytotoxic chemotherapy regimens, such as FOLFIRINOX, gemcitabine/nab-paclitaxel, and nanoliposomal irinotecan/fluorouracil, are all associated with a survival benefit ranging from 2 to 6 months when compared with single-agent gemcitabine [4]. Immune cell-based strategies comprise a diverse spectrum of modalities, such as CAR-T cells, natural killer (NK) cell infusions, and other adoptive cell transfer technologies [24]. These approaches are designed to achieve precise targeting of tumor-associated antigens, counteract immunosuppressive elements within the TME, and establish long-lasting immunologic memory against cancer cells [25]. The mechanistic foundation encompasses both direct activation of cytotoxic immune effector functions and indirect remodeling of the tumor microenvironment via cytokine release and recruitment of endogenous immunity [26].

Since the initial FDA approval in 2017 of tisagenlecleucel (tisa-cel), a Cluster of Differentiation 19 (CD19)-directed CAR-T therapy for relapsed/refractory B-cell acute lymphoblastic leukemia and diffuse large B-cell lymphoma, cell-based therapeutics have garnered renewed and widespread attention [27,28]. To date, seven CAR-T products have received regulatory approval for hematologic malignancies, reflecting the remarkable clinical progress in this area. Nonetheless, the development of cell therapies for solid tumors, including pancreatic cancer, remains at an earlier stage, confronted by unique biological challenges [29]. This review aims to summarize the current advances in CAR-immune cell-based immunotherapy for PDAC, delving into foundational mechanistic principles, summarizing clinical progress and ongoing challenges, and offering insights into future directions. We will highlight how innovative technologies, such as gene editing, various cell engineering methodologies, and combination regimens informed by spatial transcriptomics and microenvironmental mapping, are progressively redefining the therapeutic arsenal against this devastating disease. By integrating discoveries from basic immunology, clinical trials, and translational research, this article aims to provide a comprehensive overview of how immune cell-based strategies are contributing to the gradual breach of pancreatic cancer’s therapeutic barriers.

### 1.5 Review Scope and Literature Search

The primary aim of this narrative review is to comprehensively summarize and critically evaluate the current advances in chimeric antigen receptor (CAR)-immune cell-based immunotherapy for pancreatic ductal adenocarcinoma (PDAC). Specifically, this review seeks: To delineate the foundational mechanistic principles of various CAR-engineered immune cells (including T cells, NK cells, and macrophages) and their modes of action against pancreatic cancer. To synthesize the existing preclinical and clinical evidence, highlighting both the promising outcomes and the persistent challenges, such as the immunosuppressive tumor microenvironment and antigenic heterogeneity. To explore how innovative technologies, including gene editing and novel engineering methodologies, are reshaping the therapeutic landscape. By integrating insights from basic immunology, clinical trials, and translational research, this article aims to provide a foundational overview of how these sophisticated cellular strategies are progressively breaking down the formidable barriers to treating PDAC.

The literature for this narrative review was identified through a systematic search of the PubMed/MEDLINE, Web of Science, and clinicaltrial.gov electronic databases. The search strategy utilized a combination of keywords and Medical Subject Headings (MeSH) terms, including: “pancreatic cancer,” “pancreatic ductal adenocarcinoma,” “CAR-T,” “CAR-M,” “CAR-NK,” “chimeric antigen receptor,” “cell therapy,” and “immunotherapy.” The search was limited to articles published in English, with a primary focus on studies from the last decade (2014–2025) to capture the most emerging data. The initial search results were screened by title and abstract for relevance to the mechanisms, clinical applications (preclinical or clinical), and future directions of CAR-engineered immune cell therapies in pancreatic cancer. Full-text articles of the selected publications were then retrieved and assessed for eligibility. Key data, including study design, target antigens, findings, and conclusions, were extracted and synthesized to form the basis of this review.

## 2 Biological Insights of TME of Pancreatic Cancer

**Figure 1:**
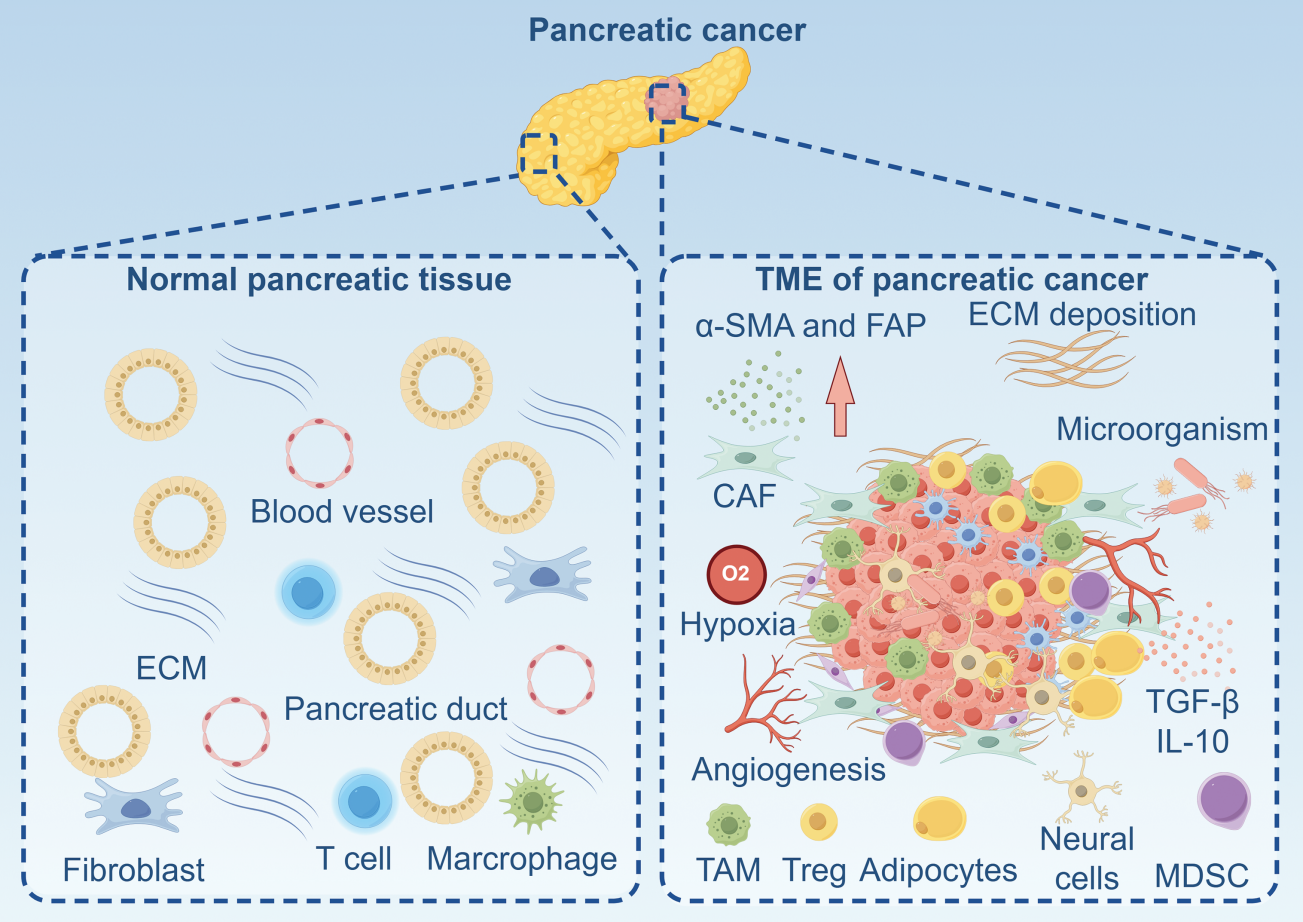
The landscape of normal pancreatic tissue and tumor microenvironment of pancreatic cancer. α-SMA: Alpha-Smooth Muscle Actin; CAF: Cancer-Associated Fibroblast; ECM: Extracellular Matrix; FAP: Fibroblast Activation Protein; IL-10: Interleukin-10; MDSC: Myeloid-Derived Suppressor Cells; TAM: Tumor-Associated Macrophage; TGF-β: Transforming Growth Factor-Beta; TME: Tumor Microenvironment; Treg: Regulatory T Cell. Created with figdraw (figdraw.com)

The biological landscape of the PDAC TME is not merely a passive backdrop but an active orchestrator of tumor progression, immune evasion, and therapeutic failure. The immune system, with its diverse cellular constituents, plays a pivotal and paradoxical role within this milieu. Infiltrating immune cells engage in a dynamic interplay with cancer cells, a vast array of stromal components, and the extracellular matrix (ECM), collectively forming a highly organized yet immunosuppressive ecosystem. This microenvironment encompasses cancer cells, a recently discovered population of intratumoral microorganisms, stromal cells such as CAFs, endothelial cells, pericytes, and neural cells, alongside a dense, remodeled ECM (Fig. 1). Understanding the nuanced biology of each component and their intricate crosstalk is fundamental to developing effective immune cell-based therapies capable of overcoming the formidable barriers posed by PDAC (Fig. 1).

### 2.1 Cancer Cell and Its Interplay with Intratumoral Microorganisms

#### 2.1.1 Cancer Cell-Intrinsic Mechanisms

Pancreatic cancer cells function not as passive targets of immune surveillance but as active architects of a highly immunosuppressive TME [30]. This cell-intrinsic reprogramming is initiated by near-universal oncogenic drivers, predominantly mutant *KRAS*, which serves as a master regulator of immune evasion in PDAC [30]. *KRAS* mutations occur in approximately 95% of PDAC cases [31] and orchestrate a pro-tumorigenic crosstalk by promoting the sustained deposition of a dense, fibrotic stroma and secreting an array of soluble factors. Critical among these is granulocyte-macrophage colony-stimulating factor (GM-CSF), which facilitates the differentiation and recruitment of immunosuppressive myeloid-derived suppressor cells (MDSCs), along with chemokines such as CXCL1, CXCL2, and CXCL5 that further enhance myeloid cell infiltration [32]. Additionally, PDAC cells employ multiple immune escape strategies, including downregulation of MHC-I expression, reduction of Fas receptors, and induction of T cell apoptosis. They also recruit regulatory T cells (Tregs) and secrete immunosuppressive cytokines like interleukin (IL)-10 and transforming growth factor-beta (TGF-β) to inhibit effector T cell function [33]. Furthermore, immune evasion in PDAC is exacerbated by profound metabolic adaptations within the TME, characterized by nutrient deprivation due to avid glucose consumption and accumulation of immunosuppressive metabolites such as lactate and kynurenine, which collectively impair and suppress anti-tumor immune effector cells [34,35].

#### 2.1.2 Multifaced Roles of Intratumoral Microorganisms

Beyond these cancer cell-intrinsic mechanisms, the TME of PDAC is further modulated by microbial influences. Once believed to be sterile, the pancreas is now recognized to host a specific microbiome, with pancreatic tumors demonstrating a significantly enriched microbial community compared to healthy or adjacent normal tissue [36,37]. This colonization reflects a dynamic and synergistic relationship with cancer cells rather than a passive presence. Certain bacterial species, such as Gammaproteobacteria including Pseudomonas and Klebsiella, directly contribute to chemoresistance by metabolizing the chemotherapeutic agent gemcitabine into its inactive form, thereby enhancing tumor cell survival [38]. In the *KrasG12D/Pdx1-Cre* mouse model, antibiotic treatment effectively inhibits pancreatic microorganisms and inhibits the accumulation of MDSCs, promoting Th1 differentiation, M1 macrophage polarization, and activation of CD4^+^ and CD8^+^ T cells [39]. Concurrently, microbial components within the tissue selectively activate Toll-like receptors (TLRs), steering monocyte differentiation toward immunosuppressive phenotypes and facilitating T cell tolerance, ultimately accelerating tumor progression [40]. Pushalkar et al. demonstrated, using a human fecal microbiota transplantation mouse model, that microbiota from long-term survivors of pancreatic cancer can inhibit tumor growth by remodeling the intratumoral microbial composition, enhancing CD8^+^ T cell activation, and suppressing the accumulation of MDSCs and regulatory T cells [41]. Furthermore, specific intratumoral microorganisms influence disease progression through immune modulation. *Fusobacterium nucleatum* infiltrates tumors via the CXCL1-CXCR2 axis-induced MDSCs, reduces CD8^+^ T cell infiltration, and accelerates tumor growth [42]. *Porphyromonas gingivalis* promotes tumor progression by stimulating neutrophil chemokine production, recruiting neutrophils, and inducing elastase secretion [43]. The oral and gut microbiota also play a role in shaping the immune landscape of pancreatic tumors [44,45]. In antibiotic-treated mice, fecal microbiota transplantation significantly restored microbial abundance in pancreatic tissue and altered the local distribution of macrophages, neutrophils, and CD8^+^ T cells, indicating that microbial transfer can regulate the tumor microbiota and reshape the immune microenvironment in pancreatic cancer [46].

### 2.2 Stromal Barrier

The pancreatic tumor microenvironment is distinguished by an exceptionally dense and complex stromal compartment that forms a formidable physical and functional barrier against immune cell-mediated antitumor responses. This fibroinflammatory stroma, frequently accounting for up to 90% of the tumor volume, is not a passive component but an active contributor to tumor progression, immune evasion, and therapeutic resistance [47]. Central to the formation and maintenance of this stromal barrier are CAFs and their principal cellular precursors, pancreatic stellate cells (PSCs). In the healthy pancreas, PSCs remain in a quiescent state, localized in periacinar and periductal regions and characterized by the presence of vitamin A-containing lipid droplets [48]. However, upon exposure to various tumor-derived factors, such as TGF-β, platelet-derived growth factor (PDGF), IL-1, and reactive oxygen species, PSCs undergo pronounced activation and phenotypic transformation [49]. During pancreatic cancer progression, static PSCs are activated by multiple stimuli, including IL-1, IL-6, hypoxia-inducible factor 1α (HIF1α), and TGF-β, evolving into a myofibroblast-like phenotype [50]. These activated PSCs exhibit increased proliferative capacity and facilitate cancer progression through the secretion of diverse cytokines. Moreover, PSCs promote fibrosis via several signaling pathways, such as IL-6, Sonic Hedgehog (SHH), vitamin D receptor (VDR), and the CXCL12/CXCR4 axis [51]. Recent evidence indicates that transcriptional regulation involving super enhancers (SE) plays a critical role in PSC activation; inhibition of SE activity can suppress genes associated with PSC activation and revert the activated phenotype (a-PSC) [52]. Additionally, PSCs can differentiate into CAFs, which subsequently produce ECM components and modulate PDAC progression and treatment response [53,54]. Thus, PSCs are pivotal regulators of both pancreatic cancer development and therapeutic outcomes.

CAFs in PDAC exhibit complex origins and differentiation pathways, significantly driving tumor-associated desmoplasia. These cells actively remodel the ECM, facilitating cancer cell invasion and metastasis [18]. They enhance the expression of matrix metalloproteinases (MMPs), which promote ECM degradation and invasion, and contribute to tissue stiffness through cross-linking and matrix remodeling, ultimately inducing hypoxia and fostering a more aggressive tumor phenotype. CAFs are highly heterogeneous and consist of functionally distinct subsets, most notably myofibroblastic CAFs (myCAFs) and inflammatory CAFs (iCAFs) [55]. myCAFs, typically located near tumor cells and driven by TGF-β signaling, are chiefly responsible for excessive ECM deposition and contraction [56]. In contrast, iCAFs, situated farther from cancer cells and influenced by IL-1 and JAK/STAT signaling, function as prolific secretory cells that release a variety of cytokines and chemokines to shape the immune landscape [57]. A key feature of CAFs is their expression of fibroblast activation protein α (FAPα), which exerts immunosuppressive effects within the TME; high FAP expression correlates with poor patient prognosis [58]. Furthermore, CAFs abundantly secrete C-X-C Motif Chemokine Ligand 12 (CXCL12, also known as Stromal Cell-Derived Factor 1, SDF-1), establishing a chemokine gradient around cancer cell nests [59]. This gradient sequesters CXCR4-expressing cytotoxic T lymphocytes (CTLs) away from tumor cells while recruiting regulatory T cells (Tregs), thereby amplifying local immunosuppression [60]. CAFs also produce other immunosuppressive factors such as IL-6, which skews T-cell polarization toward Th2 and inhibits dendritic cell maturation; galectin-1, which induces T-cell apoptosis; and leukemia inhibitory factor (LIF), which is linked impaired antitumor immunity [61]. Expression of immune checkpoint ligands like Programmed Cell Death Ligand 1 (PD-L1) and FAP on CAFs further directly inhibits T-cell function and presents potential therapeutic targets [62]. The metabolic environment within the stroma-rich TME also poses a major challenge to immune function. Extensive fibrosis exacerbates hypoxia, prompting upregulation of hypoxia-inducible factors (HIFs) in both malignant and stromal cells [63]. Consequently, accumulation of adenosine in the TME suppresses T-cell and NK cell activity while expanding Treg populations, adding another dimension of metabolic immunosuppression to the existing physical and cytokine-mediated barriers [64,65].

### 2.3 Immune Cell Heterogeneity

T cells and regulatory T cells (Tregs) play a central role in orchestrating the immune landscape of pancreatic cancer. Infiltration levels of T lymphocytes, particularly CD4+ effector T cells and cytotoxic T lymphocytes (CTLs), serve as critical independent prognostic indicators in PDAC, with robust CTL infiltration associated with improved patient survival. Nonetheless, pancreatic cancer cells deploy multiple mechanisms to evade immune destruction, including the secretion of immunosuppressive cytokines and downregulation of major histocompatibility complex class I (MHC I), thereby impairing CD8+ T cell activation and antigen recognition [66]. The death receptor Fas (CD95) also contributes significantly to immune escape; reduced Fas expression on tumor cells confers resistance to apoptosis, while cancer cells themselves engage the Fas/FasL pathway to induce apoptosis in CD8+ T cells [67]. Consequently, dysregulated Fas signaling reinforces both tumor survival and local immunosuppression, and higher Fas expression correlates with improved patient outcomes. CD4+CD25+ Tregs are often abundant among tumor-infiltrating lymphocytes and drive potent immunosuppression through the secretion of IL-10 and TGF-β, which inhibit effector T cell function and promote tumor progression and metastasis [68]. High Treg density is strongly associated with advanced disease and poor prognosis [69]. Notably, targeting tumor necrosis factor receptor 2 (TNFR2)-expressing Tregs with a monoclonal antibody has been shown to reduce markers of T cell exhaustion in CD8+ T cells. Combining anti-TNFR2 with an agonistic anti-CD40 antibody enhances T cell activation, suppresses tumor growth, and improves survival and immunological memory in murine PDAC models [70].

Tumor-associated macrophages (TAMs), especially those polarized to an M2 phenotype, constitute a major immune population within the TME and facilitate tumor growth, invasion, and metastasis. Polarized by cancer-derived factors such as M-CSF, IL-4, IL-10, and TGF-β, M2 TAMs suppress antitumor immunity, stimulate angiogenesis, and promote ECM remodeling [71]. Additionally, TAMs stimulate pancreatic stellate cell (PSC) proliferation and ECM secretion via TGF-β1 and PDGF, thereby exacerbating fibrosis and influencing tumor cell motility and vascular permeability [72]. Early during tumorigenesis, pancreatic cancer cells secrete CCL2, recruiting monocytes and macrophages through C-C Motif Chemokine Receptor 2 (CCR2)/CCR4 signaling. This recruitment is perpetuated via a positive feedback loop involving TGF-β and IL-10, with CAFs further contributing to C-C Motif Chemokine Ligand 2 (CCL2) production in advanced disease [73]. TAMs are also linked to squamous subtype gene downregulation, adenosine-mediated immunosuppression, and activation of immunogenic gene programs, potentially enabling transitions between PDAC subtypes [74,75].

MDSCs represent another crucial immunosuppressive component within the PDAC TME. This heterogeneous group of immature myeloid cells suppresses T cell function through multiple mechanisms, including the production of arginase-1, inducible nitric oxide synthase (iNOS), IL-10, and cyclooxygenase-2 (COX2) [76]. IL-10-induced Signal Transducer and Activator of Transcription 3 (STAT3) phosphorylation upregulates immune checkpoints such as PD-L1 and Cytotoxic T-Lymphocyte-Associated Protein 4 (CTLA-4) on MDSCs, further inhibiting T cell activation [77]. MDSCs also engage in cross-talk with other immune cells: they drive M2 macrophage polarization, impair antigen presentation via MHC II downregulation, promote N2 neutrophil polarization, and inhibit dendritic cell function, collectively reducing interferon (IFN)-γ production by T cells [78]. Circulating MDSC levels correlate with disease stage in PDAC patients [79], and pancreatic stellate cells enhance MDSC differentiation through STAT3-dependent chemokine secretion [80]. Furthermore, tumor-derived GM-CSF fosters MDSC expansion, reinforcing an immunosuppressive milieu [81]. Given that elevated MDSC abundance is linked to tumor progression and poor prognosis, targeting these cells represents a promising therapeutic strategy [82].

### 2.4 Other Cell Components in TME

Finally, other cellular components within the TME, including adipocytes, endothelial cells, pericytes, and neural cells, contribute significantly to its immune-suppressive character and structural complexity [83]. Adipocytes constitute a significant element within the PDAC TME and contribute to tumor progression. Prolonged exposure to cancer cells induces adipocytes, particularly those from obese individuals, to undergo phenotypic changes entailing lipid depletion and acquisition of fibroblastic or myofibroblast characteristics, thereby expanding the pool of CAFs [84]. Adipocytes secrete adipokines, pro-inflammatory cytokines, and fatty acids that support cancer cell growth, metabolic adaptation, invasion, and therapy resistance [85,86]. In turn, tumor-derived exosomes and factors like TNF-α and microRNAs alter adipocyte behavior, establishing a feed-forward loop that accelerates malignancy [87,88]. Targeting adipocyte-tumor cell crosstalk may thus offer novel therapeutic opportunities [89]. Neural components also play key roles in PDAC progression. Perineural invasion is associated with neural remodeling and hypertrophy, and vagally derived acetylcholine has been shown to reprogram the immune landscape by suppressing CD8+ T-cell recruitment through epigenetic downregulation of CCL5, inhibiting IFNγ production, and polarizing T-cell responses toward a Th2-dominant state [90]. Schwann cells further modulate the TME by interacting with macrophages, dendritic cells, mast cells, and MDSCs, reinforcing an immunosuppressive niche [91–93]. Additionally, endothelial cells and pericytes demonstrate altered phenotypes in PDAC. Cancer-derived exosomes induce pericytes to express high levels of alpha-Smooth Muscle Actin (αSMA), resulting in abnormal biomechanical and immunomodulatory properties that exacerbate hypoxia and immunosuppression [94]. Tumor influences also reduce Platelet Endothelial Cell Adhesion Molecule 1 (PECAM1) expression on endothelial cells while elevating P-selectin and ICAM1. Pericytes additionally exhibit upregulated CD274, P-selectin, and E-selectin, implicating them in vascular dysfunction and immune evasion [95].

## 3 CAR-Immune Cell Therapy Platforms in Pancreatic Cancer: Classifications and Mechanisms

Over the past decade, adoptive cell therapy (ACT) has emerged as a transformative strategy in cancer treatment by harnessing the immune system’s capacity to precisely recognize and eliminate malignant cells [96,97]. A prominent example is CAR-cell therapy, which entails genetically engineering immune cells to express a synthetic receptor that directs them to specific tumor-associated antigens [98]. CAR-T cell therapies, in particular, combine the antigen-targeting precision of antibodies with the potent cytotoxic mechanisms of T cells, leading to remarkable clinical success in hematologic malignancies and marking a revolutionary advance in the field of immunotherapy [99]. However, extending these achievements to solid tumors such as PDAC has proven challenging, primarily due to the immunosuppressive TME and inadequate T cell infiltration [100,101].

A typical CAR construct comprises several modular components: an extracellular antigen-binding domain, a hinge and transmembrane region, and intracellular signaling modules together with co-stimulatory elements [26]. The evolution of CAR design has progressed through several generations, each enhancing functional potency and persistence. First-generation CARs contained only a single intracellular signaling domain, which proved insufficient for sustained T cell activation and antitumor efficacy. This limitation spurred the development of second-generation CARs, which incorporate co-stimulatory domains such as CD28 or 4-1BB to improve T cell expansion, persistence, and cytotoxicity [102]. Third-generation CARs further amplify signaling by combining multiple co-stimulatory domains. Fourth-generation CARs, also termed “TRUCKs” (T cells redirected for universal cytokine killing), are engineered to secrete immunomodulatory cytokines (e.g., IL-12, IL-15) or express co-stimulatory ligands, thereby reshaping the TME and augmenting overall immune reactivity [103]. Most recently, fifth-generation CARs have been developed, which feature truncated cytokine receptor subunits (e.g., from IL-2R) fused to STAT transcription factor binding motifs. These advanced constructs not only enhance CAR-T cell expansion and survival but also potentiate broader immune activation by recruiting endogenous immune pathways [104,105]. Therefore, promising strategies including CAR-T, CAR-natural killer (CAR-NK) cells and CAR-macrophage (CAR-M) therapies are being explored in preclinical and clinical settings against PDAC.

### 3.1 CAR-T Therapy against Pancreatic Cancer

#### 3.1.1 Basic Principle and Advantages of CAR-T in Pancreatic Cancer

The fundamental principle of CAR-T technology involves the genetic modification of a patient’s own T lymphocytes to express a synthetic receptor that combines an extracellular antigen-binding domain, typically a single-chain variable fragment (scFv) derived from a monoclonal antibody, with intracellular T-cell signaling and costimulatory domains [106]. This engineering allows T cells to recognize and eliminate tumor cells in a major histocompatibility complex (MHC)-independent manner, thereby bypassing a key mechanism of tumor immune evasion. In the context of pancreatic cancer, the theoretical advantages of CAR-T therapy are significant. PDAC is characterized by a profoundly immunosuppressive TME, dense fibrotic stroma (desmoplasia), and a low mutational burden that contributes to its resistance to conventional therapies and immune checkpoint inhibitors [30319627]. CAR-T cells offer the potential for highly specific, potent, and adaptable immune targeting. They can be designed to attack tumor cells expressing specific tumor-associated antigens (TAAs), such as claudin 18.2, muc1, mesothelin (MSLN), carcinoembryonic antigen (CEA), human epidermal growth factor receptor 2 (HER2), or prostate stem cell antigen (PSCA), which are frequently overexpressed in PDAC [107–109]. Furthermore, unlike bispecific antibodies or adoptive T-cell therapies reliant on endogenous T-cell receptors, CAR-T cells are engineered for enhanced potency and can undergo clonal expansion and persistence *in vivo*, potentially conferring long-term immunosurveillance. This is a critical advantage for a cancer with a high rate of recurrence [110].

#### 3.1.2 Construct Design and Cell Sources of CAR-T

**Figure 2:**
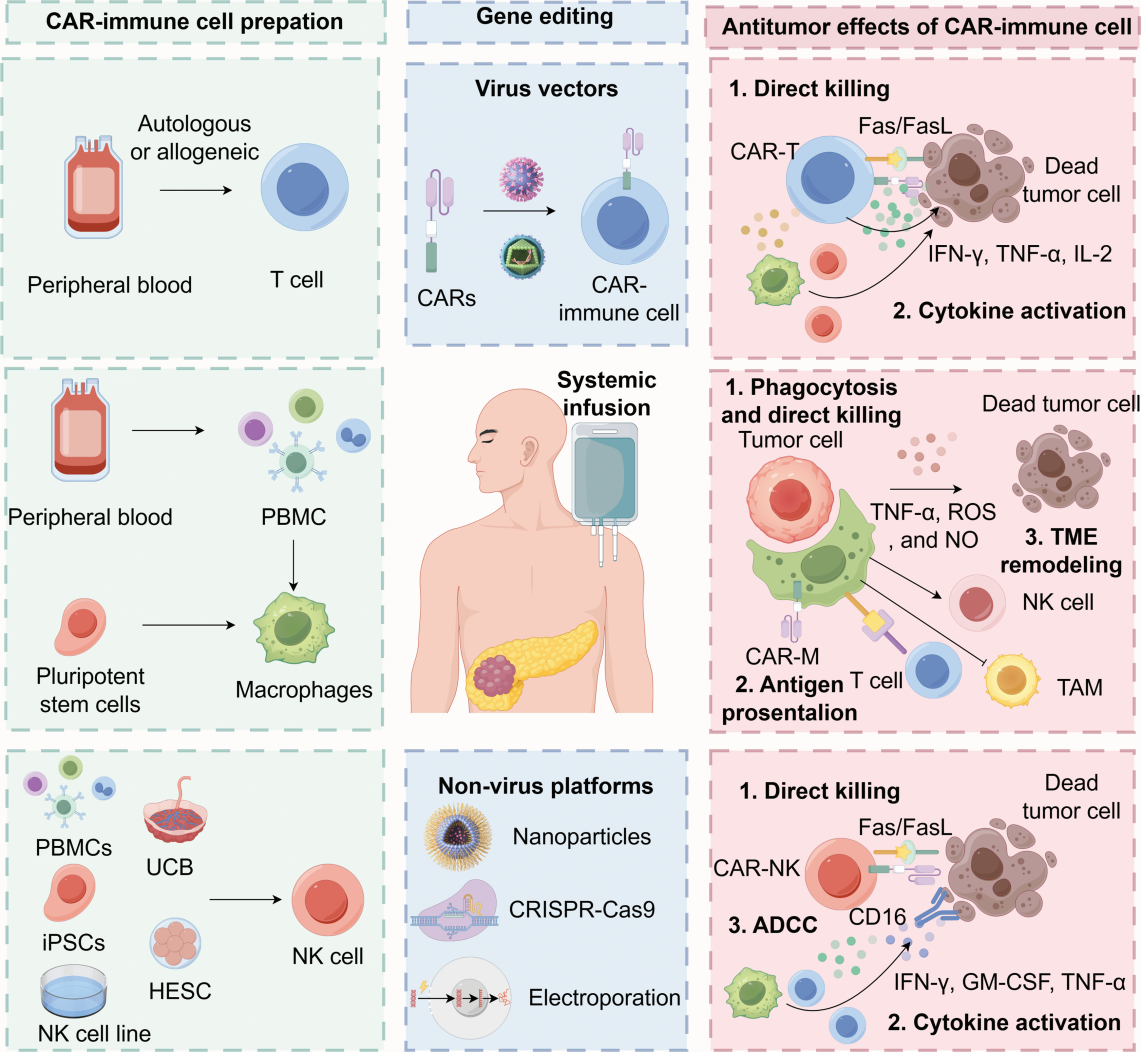
The sources, editing technologies, and anti-tumor effects of CAR-T, CAR-M, and CAR-NK in cancer immunotherapy. CAR: Chimeric Antigen Receptor; CAR-NK: Chimeric Antigen Receptor Natural Killer Cell; CAR-T: Chimeric Antigen Receptor T-cell; CRISPR-Cas9: Clustered Regularly Interspaced Short Palindromic Repeats and CRISPR-associated protein 9; Fas: First apoptosis signal; FasL: Fas Ligand; GM-CSF: Granulocyte-Macrophage Colony-Stimulating Factor; HESC: Human Embryonic Stem Cell; IFN-γ: Interferon-gamma; IL-2: Interleukin-2; iPSC: Induced Pluripotent Stem Cell; NK: Natural Killer Cell; NO: Nitric Oxide; PBMC: Peripheral Blood Mononuclear Cell; ROS: Reactive Oxygen Species; TAM: Tumor-Associated Macrophage; TNF-α: Tumor Necrosis Factor-alpha UCB: Umbilical Cord Blood. Created with figdraw (figdraw.com)

The design of the CAR construct is a critical determinant of therapeutic efficacy, safety, and persistence. First-generation CARs, which contained only a CD3ζ signaling domain, demonstrated limited expansion and clinical effectiveness [111]. Subsequent generations have incorporated one or more costimulatory domains, such as CD28 or 4-1BB (CD137), which significantly enhance T-cell proliferation, cytokine secretion, persistence, and resistance to exhaustion. For solid tumors like PDAC, third and fourth-generation CARs (often termed Tandem or TRUCK cells) are under intense investigation [22]. These designs incorporate multiple costimulatory signals or inducible cytokine expression systems (e.g., IL-12, IL-15) to bolster T-cell function within the hostile TME. The choice of the antigen-binding scFv is paramount; it must possess high affinity and specificity for the target TAA to minimize on-target, off-tumor toxicity. This is a particular concern in PDAC, as many candidate TAAs (e.g., MSLN) exhibit low-level expression on normal tissues. To address this, strategies employing logic-gated CAR systems (e.g., AND, NOT gates) and tunable activation switches are being developed to improve tumor specificity [112]. The primary cell source for CAR-T therapy is autologous T cells derived from the patient’s own peripheral blood mononuclear cells (PBMCs) (Fig. 2) [113]. While this approach avoids allorejection, it faces significant limitations in PDAC. Patients are often severely lymphopenic due to previous cytotoxic therapies or the cachectic effects of the cancer itself, making leukapheresis and adequate T-cell collection difficult [114]. Furthermore, these T cells may be functionally compromised or exhausted. Allogeneic CAR-T cells derived from healthy donors offer an “off-the-shelf” alternative. To prevent graft-vs.-host disease (GvHD), these cells are frequently genetically edited using technologies like CRISPR/Cas9 or TALENs to disrupt the endogenous T-cell receptor (TCR) genes [115]. While promising, allogeneic approaches face challenges of host-mediated rejection and potentially shorter persistence [116]. Beyond conventional αβ T cells, other lymphocyte subsets, such as γδ T cells, which possess innate-like cytotoxicity and do not cause GvHD, are emerging as attractive alternative platforms for allogeneic CAR therapy [117].

#### 3.1.3 Antitumor Mechanisms of CAR-T in Pancreatic Cancer

The antitumor mechanisms of CAR-T cells in pancreatic cancer involve a multifactorial response that extends beyond direct cytotoxic effects (Fig. 2) [118]. Following antigen recognition, CAR-T cells activate and initiate a robust cytolytic program, primarily mediated through the perforin-granzyme pathway, where perforin facilitates the entry of granzymes into target cells, inducing apoptosis, as well as through death receptor signaling such as the Fas/FasL axis [119]. In addition to direct tumor cell lysis, activated CAR-T cells undergo substantial clonal expansion and release an array of pro-inflammatory cytokines and chemokines, including IFN-γ, tumor necrosis factor-alpha (TNF-α), and IL-2. This cytokine surge contributes directly to antitumor activity and modulates the TME [120]. For instance, IFN-γ can upregulate MHC expression on tumor cells, enhance antigen presentation, recruit and activate innate immune cells such as macrophages and NK cells, and suppress pro-angiogenic signaling within stromal compartments [121,122].

### 3.2 CAR-Macrophages (CAR-M) Therapy against Pancreatic Cancer

#### 3.2.1 Basic Principles and Advantages of CAR-M in Pancreatic Cancer

CAR-Ms have emerged as a promising therapeutic strategy for solid tumors, particularly PDAC, due to their innate capacity to infiltrate tumors, remodel the TME, and mediate immune-dependent tumor elimination [123,124]. Macrophages represent a major immune component in the PDAC TME (Fig. 1) and play a central role in disease pathogenesis by adopting either pro-inflammatory, antitumoral (M1-like) or immunosuppressive, pro-tumoral (M2-like) polarization states [125,126]. During early tumorigenesis, tumor-infiltrating macrophages often exhibit an M1-like phenotype and stimulate Th1-mediated antitumor immunity. With disease progression, however, they frequently polarize toward an M2-like state, secreting factors such as IL-10, TGF-β, and VEGF that foster immune evasion and tumor growth [127,128]. Elevated M2-type TAM infiltration correlates with poor prognosis in PDAC [129], highlighting the therapeutic potential of reprogramming TAMs toward an antitumor phenotype. CAR-M therapy exploits the intrinsic plasticity and tumor-homing ability of macrophages by engineering them to express CARs that direct tumor cell recognition and killing. Upon antigen engagement, CAR-Ms phagocytose target cells, sustain M1-like pro-inflammatory cytokine secretion, and stimulate adaptive immunity [130,131]. In contrast to CAR-T cells, which are often impaired in immunosuppressive TMEs, CAR-Ms remain functional, serve as antigen-presenting cells, and activate tumor-reactive T cells [132]. These advantages are especially relevant in PDAC, where T cell infiltration is limited, exhaustion is common, and overall activity is subdued, factors that also reduce the efficacy of tumor-infiltrating lymphocyte (TIL) therapy and bispecific antibodies, both of which rely on functional and abundant T cells. Macrophages, by contrast, naturally accumulate and persist in such conditions, positioning CAR-Ms as a compelling immunotherapeutic approach for PDAC [132]. Nonetheless, the clinical translation of CAR-M therapies has progressed more slowly than that of CAR-T cells, owing to challenges such as the recent development of macrophage-specific genetic engineering platforms, incomplete understanding of optimal signaling domains for CAR design in macrophages, difficulties in achieving sustained *in vivo* persistence and activity, and practical hurdles in *ex vivo* expansion and genetic modification. Macrophage plasticity within the TME also raises concerns about potential phenotypic switching. Despite these obstacles, growing preclinical and early clinical evidence supports the safety, feasibility, and therapeutic potential of CAR-Ms for solid tumors [133,134].

#### 3.2.2 Construct Design and Cell Sources of CAR-M

The CAR-M design for solid tumors like PDAC requires careful optimization. Key goals include enhancing macrophage persistence and phagocytosis, sustaining M1 polarization, effective tumor targeting, and TME remodeling to promote a robust and durable anti-tumor response.

The antigen-binding domain of CAR-Ms typically consists of a single-chain variable fragment (scFv) derived from monoclonal antibodies TAAs. Many TAAs explored in CAR-T studies for PDAC, including MSLN, CEA, epidermal growth factor receptor (EGFR), CD147, PSCA, MUC1, TROP2, and CD276, are also under investigation for CAR-M therapy [123,135]. Similar to other CAR-based strategies, CAR-Ms carry a risk of on-target off-tumor toxicity due to TAA heterogeneity or low-level expression on healthy tissues. Therefore, selecting highly tumor-specific antigens is essential to minimize such risks. Alternatively, dual-targeting CAR systems have shown promise in enhancing tumor specificity and reducing off-target effects [136]. Structurally, the hinge and transmembrane domains provide stability and anchorage. A flexible hinge region (often from CD8α or IgG4) improves antigen-binding accessibility, while the transmembrane domain (commonly derived from CD8α or CD28) integrates the CAR into the macrophage membrane and supports signal transduction. Unlike CAR-T cells, CAR-Ms frequently employ macrophage-specific intracellular signaling domains such as CD3ζ or Fc Receptor Gamma Chain (FcRγ), which trigger phagocytosis and cytokine release upon antigen engagement [130]. Some designs also incorporate innate immune adaptors like MyD88 or CD40 to augment inflammatory responses and sustain M1 activation [137]. For instance, second-generation CAR-Ms with a CD3ζ–TIR (Toll/IL-1 receptor) fusion domain exhibit enhanced antitumor activity and M1 polarization via Nuclear Factor Kappa b (NF-κB) signaling, while resisting M2 reprogramming [131]. Incorporation of co-stimulatory domains (e.g., CD28 or 4-1BB) further promotes CAR-M survival, pro-inflammatory cytokine secretion (e.g., IL-12 and TNF-α), and M1 polarization, critical functions for reversing immunosuppression in the PDAC TME [130]. To improve tumor penetration and durability, CAR-Ms can be engineered to secrete matrix-degrading enzymes such as hyaluronidase, disrupting physical barriers in the TME. Dual-targeting approaches aimed at both tumor antigens (e.g., MSLN, claudin 18.2) and stromal components (e.g., fibroblast activation protein) may further enhance efficacy [138]. Other modifications include overexpression of anti-apoptotic genes (e.g., B-Cell Lymphoma 2, BCL-2 family members) to extend survival, secretion of immunostimulatory cytokines (e.g., IL-12, IL-15, IL-18, GM-CSF) to recruit and activate endogenous immune cells, and expression of checkpoint-blocking molecules (e.g., anti-PD-L1, anti-CD47) to counteract immune evasion [139,140].

Macrophages for CAR-M therapy can be sourced from various origins, each with distinct advantages and limitations. Monocyte-derived macrophages (MDMs) are widely used due to ease of access, autologous compatibility, and well-established differentiation protocols using M-CSF or GM-CSF (Fig. 2) [141]. They exhibit superior antigen presentation but have limited expansion capacity. Induced pluripotent stem cell-derived macrophages (iPSC-Macs) offer a virtually unlimited, genetically modifiable, and scalable source with potential for off-the-shelf applications. Tissue-resident macrophages provide tissue-specific functions but are heterogeneous and difficult to isolate at scale [142]. Umbilical cord blood-derived macrophages exhibit youthful phenotypes and low immunogenicity but face scalability constraints [143,144]. Established cell lines (e.g., THP-1, RAW 264.7) ensure reproducibility and are useful for research, but their therapeutic relevance is limited by reduced functional plasticity [145,146].

Besides, efficient genetic modification of macrophages is crucial for CAR-M production. Lentiviral and adenoviral vectors are commonly used, though macrophages are inherently resistant to lentiviral transduction due to SAMHD1 [147]. This can be overcome with Vpx-packaged lentivirus or the use of chimeric adenoviral vectors (e.g., Ad5f35), which achieve high transduction efficiency and promote sustained transgene expression [148]. Non-viral methods such as lipid nanoparticle (LNP)-mediated mRNA delivery enable transient CAR expression and *in situ* macrophage engineering, facilitating off-the-shelf and local application strategies. Other techniques, including electroporation and transposon systems (e.g., Sleeping Beauty) have also been explored for macrophage genetic modification [149].

#### 3.2.3 Antitumor Mechanisms of CAR-M in Pancreatic Cancer

Unlike CAR-T cells that mainly rely on direct cytotoxicity, CAR-Ms employ multiple complementary mechanisms to combat tumors, including phagocytosis, direct killing, activation of adaptive immunity, and remodeling of the TME (Fig. 2) [130]. A hallmark of CAR-Ms is their capacity for antigen-specific phagocytosis, mediated by intracellular signaling domains such as CD3ζ or FcRγ within the CAR construct [137]. This process enables engulfment and lysosomal degradation of tumor cells, enhancing antigen presentation and sustaining phagocytic activity even in immunosuppressive conditions. CAR-Ms also induce cytotoxicity through recognition of tumor-associated antigens, triggering the release of pro-inflammatory cytokines (e.g., TNF-α), reactive oxygen species (ROS), and nitric oxide (NO), which collectively promote cancer cell death via non-apoptotic pathways [150]. This mechanism offers potential against apoptosis-resistant malignancies, though ROS-mediated off-target toxicity requires careful modulation [147].

Furthermore, CAR-Ms play a pivotal role in activating adaptive immunity. They function as antigen-presenting cells, processing and presenting tumor antigens via MHC molecules to T cells, and secrete cytokines such as IL-12, TNF-α, and GM-CSF to recruit and activate cytotoxic T cells, NK cells, and dendritic cells [151]. Particularly in solid tumors like PDAC, CAR-Ms help overcome TME-mediated immunosuppression by repolarizing tumor-associated macrophages from M2 to M1 phenotype, enhancing T-cell infiltration through chemokine secretion (e.g., CXCL9), and facilitating antibody-dependent cellular cytotoxicity (ADCC) [152,153].

Additionally, CAR-Ms modify the immunosuppressive and fibrotic TME characteristic of PDAC by secreting cytokines (e.g., IL-12, IL-15) that inhibit regulatory T cells and myeloid-derived suppressor cells, and by expressing matrix metalloproteinases (MMPs) that degrade extracellular matrix components [154]. These changes promote immune cell infiltration and may synergize with immune checkpoint inhibitors. However, sustained secretion of certain cytokines (e.g., IL-6, IL-1β) risks systemic toxicity or local inflammation. Safety strategies such as inducible caspase-9 (iCasp9) switches and controlled cytokine expression are under investigation to improve the therapeutic window [155,156].

### 3.3 CAR-NK Cell Therapy against Pancreatic Cancer

#### 3.3.1 Basic Principle and Advantages of CAR-NK in Pancreatic Cancer

NK cells represent a distinct subset of lymphocytes that play a pivotal role in the innate immune system, accounting for approximately 10%–15% of all circulating lymphocytes. They are integral to immune surveillance, efficiently identifying and eliminating virally infected and malignant cells without prior sensitization [157]. In recent years, cellular immunotherapies, particularly those employing CAR-T, have achieved remarkable success in treating hematologic malignancies [158]. However, their application in solid tumors, including PDAC, remains substantially limited due to several key challenges: limited efficacy related to tumor accessibility and heterogeneity, logistical constraints in obtaining sufficient autologous T cells, severe toxicities such as cytokine release syndrome (CRS) and neurotoxicity, and prohibitively high costs [159]. In this context, CAR-NK cells have emerged as a promising alternative, combining the innate antitumor mechanisms of NK cells with the antigen-specific targeting capability of CARs. NK cells are notably advantageous in that they can be sourced from allogeneic donors without triggering significant GvHD, thereby facilitating “off-the-shelf” therapeutic production. Furthermore, they exhibit intrinsic cytotoxicity against tumor cells through both CAR-dependent and independent mechanisms, and are associated with a more favorable safety profile, including a reduced incidence of CRS [160,161].

#### 3.3.2 Construct Design and Cell Sources of CAR-NK

A typical CAR-NK construct mirrors the modular structure of CAR-T designs, comprising an extracellular antigen-binding domain (typically a single-chain variable fragment, scFv), a hinge region for flexibility, a transmembrane domain, and intracellular signaling modules. However, optimal activation in NK cells often involves incorporating tailored signaling motifs, such as CD3ζ in combination with NK-specific costimulatory domains like 2B4 (CD244), DNAX Accessory Molecule-1 (DNAM-1), or 4-1BB, which enhance persistence, cytotoxicity, and cytokine production [162,163]. These design adaptations are critical to maximizing CAR-NK function within the immunosuppressive PDAC microenvironment [164]. NK cells for CAR engineering can be derived from multiple sources, each with distinct characteristics. Peripheral blood (PB) provides mature and functionally competent NK cells, though often in limited numbers. UCB serves as a rich reservoir of less-differentiated NK cells with high expansion potential [165]. Human embryonic stem cells (hESCs) and induced pluripotent stem cells (iPSCs) offer virtually unlimited scalability and genetic manipulability, supporting the generation of standardized, clinically relevant NK cell products [166]. Additionally, established NK cell lines, such as NK-92, provide a consistent and expandable cellular platform, albeit often requiring irradiation before infusion due to their tumorigenic origin (Fig. 2) [167].

#### 3.3.3 Antitumor Mechanisms of CAR-NK in Pancreatic Cancer

Upon engagement with tumor-associated antigens, CAR-NK cells elicit potent antitumor responses through multiple mechanisms (Fig. 2). They directly mediate target cell lysis via the release of perforin and granzymes and through death receptor pathways such as FAS/FASL and TRAIL/DR5 [168]. Moreover, CAR activation enhances native NK cell functions, including ADCC mediated via CD16 (FcγRIII), and secretion of immunomodulatory cytokines such as IFN-γ, GM-CSF, and TNF-α. These cytokines not only directly inhibit tumor growth but also recruit and activate other immune cells, including dendritic cells, macrophages, and endogenous T cells, thereby fostering a more inflammatory and antitumorigenic microenvironment [169,170]. This multimodal activity is particularly valuable in PDAC, where the dense stromal barrier and immunosuppressive network pose major obstacles to T cell-centric therapies [171].

## 4 Comparison of Various CAR-T, CAR-M, and CAR-NK Therapies

The field of ACT has rapidly evolved beyond CAR-T cells to incorporate innovative platforms such as CAR-NK and CAR-M therapies, each exhibiting distinct biological mechanisms and clinical potential (Table 1). CAR-T cells, which are primarily derived from autologous or allogeneic T cells, mediate antitumor activity through MHC-restricted recognition, initiating target cell apoptosis via perforin-granzyme pathways and death receptor signaling. Although several CAR-T products, including Kymriah and Yescarta, have been approved by the FDA for hematologic malignancies, their efficacy in solid tumors such as PDAC remains limited. Key challenges include T cell exhaustion, the immunosuppressive TME, and significant risks associated with CRS and GvHD in allogeneic contexts. Additionally, the manufacturing process for patient-specific CAR-T cells is complex and costly [172].

**Table 1:** Comparison of chimeric antigen receptorT-cell (CAR-T), chimeric antigen receptor macrophage (CAR-M), and chimeric antigen receptor natural killer cell (CAR-NK) therapies

	CAR-T	CAR-M	CAR-NK
**Sources**	Autologous or allogeneic T cells from peripheral blood	Macrophages differentiated from PBMC or iPSCs	Multiple sources including PB, UCB, HESCs, IPSCs and NK cell lines
**Mechanism of action**	Direct cytotoxicity via TCR and cytokine release	Phagocytosis, antigen presentation, TME remodeling	Innate cytotoxicity, ADCC, and cytokine release
**MHC restriction**	None	None	None
**Risk of GVHD**	High	Low	Low
**Risk of CRS**	High	Potential CRS	Low
**Manufacturing Complexity**	High; gene modification and autologous expansion	High; macrophage differentiation and gene editing	Moderate; obtained from allogeneic sources with less engineering
**Cost**	High	High	Moderate
**Limitations**	Risk of GVHD, TME suppression, antigen loss, T-cell exhaustion, CRS risk, high cost	Short survival *in vivo*, scalability challenge from autologous source, pro-tumor potential	Limited persistence, moderate efficacy, potential for rejection in allogeneic settings
**FDA-approved products**	Approved for hematologic tumors	None	None
**Clinical development in PDAC**	Limited due to TME barriers and antigen heterogeneity	Early clinical trials ongoing	Early clinical trials ongoing

**Note: Abbreviation: **ADCC: Antibody-Dependent Cell-mediated Cytotoxicity; CAR: Chimeric Antigen Receptor; CAR-M: Chimeric Antigen Receptor Macrophage; CAR-NK: Chimeric Antigen Receptor Natural Killer Cell; CAR-T: Chimeric Antigen Receptor T-cell; CRS: Cytokine Release Syndrome; FDA: Food and Drug Administration; GVHD: Graft-vs.-Host Disease; HESCs: Human Embryonic Stem Cells; iPSCs: Induced Pluripotent Stem Cells; MHC: Major Histocompatibility Complex; PB: Peripheral Blood; PBMC: Peripheral Blood Mononuclear Cell; PDAC: Pancreatic Ductal Adenocarcinoma; TCR: T-cell Receptor; TME: Tumor Microenvironment; UCB: Umbilical Cord Blood.

CAR-M represent another strategy focused on phagocytosis, antigen presentation, and remodeling of the TME, rather than solely direct cytotoxicity. Generated from peripheral blood monocytes or iPSC-derived macrophages, CAR-M cells engulf tumor cells through CAR-specific signaling domains such as CD3ζ or FcRγ, and enhance adaptive immune responses via cross-presentation of antigens and secretion of cytokines like IL-12 and TNF-α. Their MHC-unrestricted targeting capability and ability to counteract immunosuppression, by repolarizing M2-type macrophages, degrading ECM components, and recruiting T cells, make them particularly promising for treating solid tumors, including PDAC. However, obstacles such as difficulties in scaling manufacturing, limited *in vivo* persistence, and unresolved concerns regarding long-term safety and cytokine-mediated toxicity (e.g., from IL-6 or IL-1β) underscore the need for further refinement. Investigational strategies include inducible safety switches (e.g., iCasp9) and *in vivo* reprogramming techniques [173,174].

In contrast, CAR-NK cells are sourced from peripheral or cord blood, established NK cell lines, or iPSCs, and function independently of MHC recognition through innate “missing-self” cytotoxicity mechanisms. Their antitumor activity involves not only perforin and death receptor pathways but also ADCC and the secretion of immunomodulatory cytokines such as IFN-γ and GM-CSF. These attributes are associated with a lower incidence of CRS and minimal risk of Graft-vs.-Host Disease (GVHD), even in allogeneic settings, supporting their development as off-the-shelf therapeutics. While no CAR-NK therapy has yet received FDA approval, early-stage clinical trials in PDAC are ongoing, motivated by their favorable safety profile and potential to modulate the TME. Nevertheless, challenges related to limited *in vivo* persistence and manufacturing difficulties in transduction and expansion efficiency remain to be addressed [175,176].

In summary, while CAR-T therapies currently lead in clinical adoption, both CAR-NK and CAR-M platforms provide complementary advantages, including enhanced safety profiles, suitability for allogeneic use, and improved potential for targeting solid tumors. Continued advances in CAR engineering, manufacturing methodologies, and combination immunotherapy regimens are poised to address existing limitations and accelerate the development of next-generation cellular therapies for refractory cancers such as PDAC.

## 5 Recent Preclinical and Clinical Advances in CAR-Immune Cell Therapy for Pancreatic Cancer

### 5.1 Recent Preclinical and Clinical Advances in CAR-T Cell Therapy for Pancreatic Cancer

#### 5.1.1 Mesothelin-Targeting CAR-T

Mesothelin is a cell surface antigen composed of two primary forms: a membrane-bound protein and a soluble fragment known as the mesothelin-related peptide (SMRP). Its encoding gene is highly upregulated in multiple malignancies, and elevated mesothelin expression correlates strongly with enhanced tumor cell proliferation, adhesion, signaling, and metastatic potential [177]. In pancreatic cancer, mesothelin is expressed in approximately 80% of patients, with 25%–100% of tumor cells exhibiting surface staining in positive cases [19]. Importantly, human T cells can be activated by MSLN-derived epitopes, effectively lysing MSLN-overexpressing tumor cells. These findings provide a compelling rationale for MSLN-directed immunotherapy as a tumor-specific strategy in pancreatic cancer [19].

Despite this promise, the clinical efficacy of MSLN-targeting CAR-T cells in PDAC remains limited due to antigen heterogeneity, a suppressive TME, and inadequate T-cell infiltration and persistence. To address these challenges, combination approaches are being intensively investigated. For example, proton radiotherapy has been shown to upregulate MSLN expression, enhance CAR-T cell trafficking, reprogram the TME toward an immunostimulatory state, and induce systemic antitumor effects, including abscopal responses [178]. Additionally, inhibition of cholesterol acyltransferase 1 (ACAT-1) was reported to potentiate the activity of MSLN-CAR-T cells against pancreatic tumors [179]. Genetic engineering strategies, such as dual knockout of Inhibitor of DNA Binding 3 (ID3) and SRY-Box Transcription Factor 4 (SOX4) in MSLN-CAR-T cells, have also been shown to prolong relapse-free survival, indicating a potential clinical activity promising for enhancing CAR-T cell potency [180]. Similarly, oncolytic viruses, including MSLN-expressing HSV or cytokine-armed adenoviruses (e.g., encoding TNF-α and IL-2), can enhance antigen presentation, reverse T-cell exhaustion, promote dendritic cell maturation and M1 macrophage polarization, and synergize with CAR-T therapy to achieve durable tumor control and suppression of metastasis [181,182]. Beyond conventional αβ T cells, alternative immune effector populations such as γδ T cells and Natural Killer T cell (NKT) cells exhibit potent antitumor activity when targeted with MSLN/CD3 bispecific antibodies, demonstrating reduced cytokine release and minimal toxicity, thereby representing a safer and effective alternative immunotherapeutic modality [183]. To overcome the stromal barrier and immunosuppressive TME in pancreatic cancer, mesoFAP CAR-T-cell-engaging molecule (TEAM) cells, equipped with a mesothelin-directed CAR and a secreted T-cell engager targeting FAP, effectively eliminate both tumor cells and CAFs, demonstrating superior antitumor activity in patient-derived and murine PDAC models [138]. Together, these findings highlight the critical need for combinatorial regimens that modulate the TME and amplify both endogenous and engineered immunity targeting MSLN, offering a multifaceted strategy to improve treatment outcomes in PDAC.

Despite initial encouraging signs of safety and biological activity in early-phase trials, mesothelin-targeted CAR-T cell therapies have exhibited limited clinical efficacy in patients with PDAC (Table 2). In a Phase I study by Beatty et al., six patients with chemotherapy-refractory metastatic PDAC received repeated intravenous infusions of MSLN-specific CAR-T cells (CART-meso). While the treatment was well-tolerated without serious adverse events, and two patients achieved disease stabilization with progression-free survival of 3.8 and 5.4 months, accompanied by a notable 69.2% reduction in metabolically active liver tumor volume in one patient, objective responses were not observed [184]. Similarly, Haas et al. reported that a second-generation SS1-based CAR construct incorporating 4-1BB and CD3ζ signaling domains led to only transient CAR-T cell persistence and modest antitumor activity, despite successful trafficking to tumor sites [185]. To address these limitations, recent efforts have turned to cytokine-augmented CAR-T platforms designed to enhance T-cell expansion, infiltration, and persistence within immunosuppressive tumor microenvironments. For instance, CAR-T cells engineered to secrete IL-7 and CCL19 (7 × 19 CAR-T) demonstrate superior migratory capacity, sustained activity, and potent antitumor effects in both preclinical models and early clinical settings. Notably, one patient with advanced pancreatic cancer achieved near-complete tumor regression following intravenous administration of anti-mesothelin 7 × 19 CAR-T cells [186].

**Table 2:** Clinical trials of CAR-T therapy in pancreatic cancer with published results

Chimeric antigen receptor	Phase	Number of patients	Dosage	Persistence	Therapeutic outcomes	References
Mesothelin	I	6	0.25–8.7 × 10^6/^kg	NA	In a patient with advanced PC, anti-MSLN-7 × 19 CAR-T treatment resulted in almost complete tumor disappearance 240 days post-intravenous infusion.	[186]
Mesothelin	I	6	1–3 × 10^8^/m^2^	Transient	Disease stabilized in 2 patients, with progression-free survival times of 3.8 and 5.4 months.	[184]
Mesothelin	I	5	1–3 × 10^7^, 1–3× 10^8^/m^2^	Up to 1 month	Disease stabilized in 3 patients.	[185]
Claudin18.2	I	5	0.5–55 × 10^8^ cells	NA	One patient achieved PR.	[195]
Claudin18.2	I	3	2.5 × 10^8^ cells	28 days	ORR was 22.2% and DCR 66.7%, with mPFS of 2.6 months.	[197]
Claudin18.2	I	2	2.5 × 10^8^ cells	NA	Two patients achieved PR or CR following CT041 treatment.	[196]
Claudin18.2	Ib	5	2.5–4 × 10^8^ cells	NA	2 had stable disease with tumor shrinkage and 3 had progression of disease.	[198]
Claudin18.2	I	1	1.2 × 10^6^ cells/kg	NA	The patient achieved a CR 1 month after therapy, and remained in clinical remission for 8 months.	[224]
EGFR	I	16	3.48 × 10^6^/kg	Up to 1 month	Of 14 evaluable patients, four achieved partial response for 2–4 months, and eight had stable disease for 2–4 months.	[210]
HER2	I	2	2.1 × 10^6^/kg	Up to 3 months	Two patients achieved SD with the PFS 5.3 and 8.3m	[208]
PSCA	I	24	2.5 × 10^6^ cells/kg	up to 250 days	12 patients achieved SD with the DCR 60%	[213]
CEA	I	1	1 × 10^10^ cells	NA	Complete metabolic response	[216]

**Note: **CR: Complete Response; CAR: Chimeric Antigen Receptor; CEA: Carcinoembryonic Antigen; DCR: Disease Control Rate; EGFR: Epidermal Growth Factor Receptor; HER2: Human Epidermal Growth Factor Receptor 2; mPFS: median Progression-Free Survival; NA: Not Applicable; ORR: Objective Response Rate; PC: Pancreatic Cancer; PR: Partial Response; PSCA: Prostate Stem Cell Antigen; SD: Stable Disease.

#### 5.1.2 Claudin18.2-Targeting CAR-T

Claudin18.2 (CLDN18.2), a member of the Claudin protein family located predominantly on cellular membranes, exhibits minimal expression in normal gastric mucosal epithelium under physiological conditions [187]. However, its expression is significantly upregulated in multiple malignancies, including approximately 80% of gastrointestinal adenocarcinomas, 60% of pancreatic tumors, as well as subsets of esophageal, ovarian, and lung cancers [188], rendering it a highly attractive therapeutic target. Preclinical studies have demonstrated promising antitumor efficacy of CLDN18.2-targeting CAR-T cells in gastric cancer [189], though their clinical applicability in pancreatic cancer remains under investigation (Table 2) [190].

Recently, sequential infusion of FAP-targeted CAR-T cells followed by CLDN18.2-specific CAR-T cells has been shown to deplete cancer-associated fibroblasts, reduce MDSC recruitment, and enhance T-cell persistence and antitumor activity [191]. In a complementary approach, engineering CLDN18.2-CAR-T cells to co-express CXCR4 enhanced tumor trafficking via the SDF-1α/CXCR4 axis and suppressed MDSC migration through modulation of the STAT3/NF-κB signaling pathway [192]. Additionally, combining localized radiotherapy with CLDN18.2-CAR-T therapy improved T-cell infiltration, effector function, and M1 macrophage polarization, resulting in significantly enhanced tumor control [193]. Pharmacological inhibition of indoleamine 2,3-dioxygenase 1 (IDO1) also augmented CAR-T cytotoxicity and reduced T-cell exhaustion by mitigating kynurenine-mediated immunosuppression, an effect further amplified by fludarabine/cyclophosphamide preconditioning (PMID: 40045363). Moreover, CXCR2-modified CLDN18.2-CAR-T cells demonstrated improved tumor homing and reduced immunosuppressive macrophage and MDSC infiltration, effectively controlling both primary and metastatic PDAC [194].

As the first CLDN18.2-directed CAR-T drug, CT041 emerged in 2019 and has since shown encouraging clinical activity in digestive system tumors. In an open-label phase I trial (NCT03159819), 12 patients (7 gastric, 5 pancreatic) received CT041 infusion. Among 11 evaluable patients, one achieved complete response (CR), three had partial responses (PR; two gastric, one pancreatic), five had stable disease (SD), and two experienced progressive disease (PD), resulting in an objective response rate (ORR) of 33.3% and a median progression-free survival (mPFS) of 130 days, indicating potential benefit in advanced pancreatic and gastric cancers [195]. Further reports from trials NCT04581473 and NCT03874897 described two patients with metastatic CLDN18.2-positive pancreatic cancer who failed standard therapies but achieved PR or CR following CT041 treatment, accompanied by manageable cytokine release syndrome (CRS) and immune cell subset changes [196]. Interim results from a phase I study (NCT03874897) involving 37 patients with metastatic gastrointestinal cancers, including five with pancreatic cancer, showed an ORR of 48.6% and disease control rate (DCR) of 73.0% overall. In the pancreatic cancer subgroup (n = 9), ORR was 22.2% and DCR 66.7%, with mPFS of 2.6 months [197]. Another phase Ib trial (NCT04404595) reported stable disease with tumor reduction in two pancreatic cancer patients among 11 participants [198]. Ongoing studies, such as NCT05911217, continue to evaluate CT041. The studies above have demonstrated that CT041, a Claudin18.2-targeted CAR-T cell drug, has good anti-tumor activity in advanced gastrointestinal tumors, including pancreatic cancer, and is safe and reliable.

Beyond CT041, several other CLDN18.2-targeted CAR-T constructs are under clinical evaluation. These include LY011 (third-generation CAR-T, NCT04977193 and NCT04966143), LB1908 (NCT05539430), HEC-016 (NCT05277987), KD-496 (NCT05583201), and CT048 (NCT05393986), though results have not yet been reported.

#### 5.1.3 HER2-Targeting CAR-T

Human epidermal growth factor receptor 2 (HER2), a transmembrane glycoprotein belonging to the epidermal growth factor receptor family, plays a critical role in regulating cell proliferation and differentiation during embryonic development and in adult tissues [199]. Amplification and overexpression of the HER2 gene and its protein product are frequently observed in multiple cancer types and are associated with more aggressive tumor behavior and poorer clinical outcomes [200]. In pancreatic and biliary tract malignancies, HER2 overexpression is detected in approximately 7%–58% and 20%–70% of cases, respectively, making it a compelling molecular target for CAR-T therapy in pancreatic cancer [201].

Ongoing advances in HER2-directed CAR-T therapy are being achieved through innovative engineering and multimodal strategies. For instance, HER2-CAR-T cells engineered to secrete IL-7 and CCL19 (referred to as 7 × 19 CAR-T) have demonstrated potent antitumor activity, including complete regression of autologous patient-derived organoids and prolonged survival in preclinical models, while circumventing allogeneic immune reactions through autologous T-cell generation [202,203]. To address on-target off-tumor toxicity, a switchable CAR-T system has been developed that enables precise control of T-cell activity via exogenous antibody-based switches, achieving complete remission in highly aggressive metastatic PDAC patient-derived xenograft models without loss of antitumor efficacy [204]. Other combinatory strategies, such as dual-targeting CARaMEL T cells directed against both HER2 and the gp100 antigen via an exogenous T-cell receptor, when coupled with vaccinia virus-based vaccination and panobinostat, promote robust memory T-cell differentiation and sustained tumor eradication in immunocompetent and xenograft models [205]. Furthermore, the utilization of specialized T-cell subsets, such as CD161+CD8+ T cells, for HER2-CAR construction enhances cytotoxic capacity, reduces exhaustion, and improves tumor control in PDAC xenografts [206]. Lastly, a modular CAR platform (P329G-CAR) designed to engage tumor-specific antibodies (e.g., anti-HER2) bearing silenced Fc domains exhibits high target specificity, prevents off-target activation, and mediates potent antitumor activity both *in vitro* and *in vivo* [207].

In an early-phase clinical trial involving 11 patients with HER2-positive (expression >50%) pancreatic or biliary tract cancers, administration of 1–2 cycles of HER2 CAR-T cells following preconditioning with nab-paclitaxel and cyclophosphamide resulted in partial remission in one patient, stable disease in five, and progressive disease in the remaining five individuals. The treatment was generally well-tolerated, though notable adverse events included significant upper gastrointestinal bleeding, fever, and elevated transaminase levels in a subset of patients [208]. Overall, these findings support the safety and clinical feasibility of HER2-targeted CAR-T cell immunotherapy in this challenging patient population. Inconsistent reporting of response duration across trials remains a challenge in the field and that standardized reporting of this key efficacy metric is crucial for accurately evaluating the long-term potential of CAR-T therapies in solid tumors.

#### 5.1.4 Other Promising Molecule-Targeting CAR-T

For other promising targets of pancreatic cancer, the development of CAR-T therapy is focusing on multiple tumor-associated antigens. EGFR is overexpressed in most PDAC, but its wide distribution in normal tissues limits the therapeutic window of CAR-T [209]. Preclinical models have shown that the combination of EGFR inhibitors can enhance specificity and delay tumor growth. A phase I trial demonstrated that EGFR-targeted CAR-T cell therapy, following lymphodepletion chemotherapy, exhibited a manageable safety profile and induced clinical responses, including partial remission and stable disease, in patients with metastatic pancreatic carcinoma, albeit with limited response duration [210].

PSCA Targeting CAR-T

PSCA is a cell surface protein that is linked to the cell membrane through a glycosylphosphatidylinositol (GPI) linkage. It is composed of 123 amino acids and has high expression levels in the majority of pancreatic tumor cells, while being expressed at lower levels in normal tissue cells [211]. Preclinically, PSCA-directed CAR-T cells equipped with an inverted cytokine receptor (4/7 ICR), designed to convert immunosuppressive IL-4 into a pro-proliferative signal via an IL-7R endodomain, demonstrate enhanced persistence and antitumor efficacy in IL-4-rich tumor microenvironments, addressing a key mechanism of immune resistance [212]. Clinically, a phase I trial evaluating the switch-activated PSCA-targeting GoCAR-T product BPX-601, which incorporates an inducible MyD88/CD40 costimulatory switch activated by rimiducid, revealed evidence of T-cell expansion, tumor infiltration, and pharmacodynamic immune activation; however, the trial was terminated due to dose-limiting toxicities and two treatment-related deaths at the highest dose level, underscoring the challenges in balancing efficacy and safety with inducible systems in heavily pretreated metastatic PDAC patients [213].

CEA Targeting CAR-T

Carcinoembryonic antigen (CEA), a classic tumor marker, is overexpressed in over 80% of pancreatic cancers, but it also faces the challenge of being expressed at low levels in normal tissues such as the colon and lungs. Preclinical models demonstrate that CEA-specific CAR-T cells can mediate significant tumor regression and long-term eradication without inducing autoimmunity against normal CEA-expressing tissues, even in the absence of lymphodepletion [214]. However, therapeutic efficacy is highly dependent on CEA expression levels, with only high-expressing PDAC models showing robust responses, highlighting the importance of patient stratification based on antigen density [215]. To overcome barriers such as poor T-cell trafficking and immunosuppressive microenvironments in solid tumors, locoregional delivery strategies have been developed. In a clinical case report, hepatic arterial infusion (HAI) of CEA-CAR-T cells using pressure-enabled drug delivery (PEDD) technology resulted in complete metabolic response in liver metastases from PDAC, accompanied by significant intratumoral CAR-T infiltration and a favorable safety profile, supporting further investigation into regional CAR-T administration for advanced disease [216].

MUC1 Targeting CAR-T

The cell surface protein known as MUC1, which comprises a sequence of 1225 amino acids, is frequently observed to be upregulated in various types of solid tumors, such as pancreatic cancer [217]. CAR-T cells engineered to recognize the cancer-specific Tn-glycoform of MUC1 exhibit potent antitumor activity in xenograft models of pancreatic cancer, demonstrating the potential of targeting glycosylation-dependent epitopes to achieve tumor selectivity [218]. However, significant heterogeneity exists in the sensitivity of PDAC cells to tMUC1-CAR-T-mediated cytolysis, with resistant lines such as HPAFII exhibiting enhanced type I and II IFN signaling and subsequent upregulation of immune checkpoints (e.g., PD-L1) and chemokines (e.g., CXCL10) upon CAR-T engagement. Inhibiting JAK1/2 with ruxolitinib or blocking PD-L1 and CXCL10 can restore CAR-T cytotoxicity, underscoring the role of tumor-autonomous IFN pathways in immune evasion [219]. Further studies identify additional resistance mediators, including IDO1, COX1/2, and galectin-9, whose pharmacological inhibition synergizes with tMUC1-CAR-T cells to enhance killing of refractory PDAC models [220].

CD133 Targeting CAR-T

CD133, a transmembrane glycoprotein overexpressed in approximately 50% of hepatocellular, pancreatic, and intrahepatic cholangiocarcinomas, has been evaluated as a target for CAR-T therapy in a phase I clinical trial involving advanced metastatic malignancies. Treatment with CD133-directed CAR-T cells resulted in manageable hematologic toxicity and preliminary antitumor efficacy, including partial responses and disease stabilization, supporting its feasibility for targeting cancer stem cells [221]. Meanwhile, CEACAM7, a cell adhesion molecule with highly restricted expression in PDAC and minimal presence in normal tissues, has emerged as a potential clinical activity target. CEACAM7-specific CAR-T cells exhibit potent antitumor activity and induce remission in patient-derived xenograft models of PDAC [222]. Preclinical investigations further demonstrate that csGRP78-targeting CAR-T cells effectively eliminate diverse pancreatic cancer cell lines and cancer stem-like cells, suppress tumor growth *in vivo*, and show enhanced activity when combined with gemcitabine through upregulation of target expression. Additionally, GAS6-directed CAR-T cells have been shown to effectively eradicate TAM-positive pancreatic cancer cell lines and significantly inhibit the growth of PANC1 xenografts and patient-derived xenografts in murine models, without inducing substantial toxicities [223].

### 5.2 Recent Preclinical and Clinical Advances in CAR-M Cell Therapy for Pancreatic Cancer

Recent advances in CAR-M therapy have unveiled promising strategies to counteract the immunosuppressive and highly fibrotic tumor microenvironment characteristic of PDAC. Preclinical studies indicate that c-MET-targeted CAR-Ms enhance phagocytosis of both tumor cells and cancer stem cells, suppress angiogenesis, and synergize with chemotherapy, significantly improving survival in murine models without observable toxicity [134,225]. Similarly, HER2-specific CAR-Ms exhibit robust antitumor efficacy, which is further potentiated by M1 polarization, resulting in elevated secretion of proinflammatory cytokines and enhanced tumoricidal activity [155]. Novel methodologies include *in vivo* reprogramming of macrophages via mannose-modified mRNA-loaded lipid nanoparticles (mRNA-LNPs) to express FAP-targeting CARs, effectively depleting cancer-associated fibroblasts and degrading collagenous barriers, thereby augmenting drug delivery and T-cell infiltration [226]. Allogeneic CAR-M platforms derived from induced pluripotent stem cells, such as PSCA-directed CAR-iMacs equipped with membrane-bound IL-15 and a safety switch, demonstrate potent antitumor responses and support off-the-shelf applicability [143]. Additional engineering strategies to disrupt the CD47–SIRPα “don’t eat me” axis through shRNA knockdown further potentiate phagocytosis, activate the cGAS-STING pathway, and stimulate adaptive antitumor immunity [227].

Building on these preclinical successes, CAR-M therapy has entered early-phase clinical trials for solid tumors, including PDAC (Table 3) [130]. A landmark achievement was the initiation of the first-in-human trial (CT-0508, NCT04660929) using autologous anti-HER2 CAR-Ms for HER2-overexpressing solid tumors. Early results indicate that CAR-M administration promotes inflammatory tumor remodeling and facilitates immune cell recruitment into immunologically cold tumors, providing a rationale for further evaluation in PDAC [228]. Ongoing clinical efforts are exploring CAR-Ms targeting other tumor-associated antigens such as EGFR and PD-L1, often in combination with immune checkpoint inhibitors or chemotherapy. By mediating antigen presentation and secreting immunostimulatory cytokines, CAR-Ms may convert immunologically excluded (“cold”) microenvironments into T-cell-infiltrated (“hot”) niches, thereby sensitizing tumors to PD-1/PD-L1 blockade. This concept is being evaluated in a current trial combining CT-0508 with pembrolizumab (anti-PD-1) in HER2-positive cancers (NCT04660929, Group 2).

**Table 3:** Ongoing clinical trials of CAR-M therapy in pancreatic cancer

Chimeric antigen receptor	Indication	Phase	Status	Number of patients	Therapeutic approach	Sponsor	Identifier
HER2	Solid tumors (including PDAC)	I	Active	48	Autologous HER2-targeting CAR-M (CT-0508)	Carisma Therapeutics	NCT04660929
HER2	Solid tumors (including PDAC)	I	Active	6	Autologous HER2-targeting CAR-M (CT-0525)	Carisma Therapeutics	NCT06254807
Mesothelin	Solid tumors (including PDAC)	NA	Recruiting	2	Targeting Mesothelin by CAR-M (SY001) + anti-PD-1	Cell Origin Biotech (Hangzhou)	NCT06562647
TROP2	Metastatic epithelial tumors (including PDAC)	I	Recruiting	48	mRNA/liquid nanoparticle delivery of CAR to *in vivo* myeloid cells (MT-302)	Myeloid Therapeutics	NCT05969041

**Note: **anti-PD-1: anti-Programmed Cell Death Protein 1; HER2: Human Epidermal Growth Factor Receptor 2; NCT: National Clinical Trial; PDAC: Pancreatic Ductal Adenocarcinoma; TROP2: Trophoblast Cell-Surface Antigen 2.

Despite this progress, the clinical translation of CAR-M therapies remains more complex than that of CAR-T cells, particularly in PDAC. A major challenge is the reliance on autologous macrophages, which presents manufacturing hurdles including limited cell yields, poor expansibility, and functional variability, especially in heavily pretreated patients. These issues are exacerbated by the aggressive natural history of PDAC, which narrows the therapeutic window and complicates patient recruitment. Furthermore, although targeting the immunosuppressive TME is a fundamental rationale for CAR-M development, this very environment may also impede therapeutic efficacy. Overcoming these barriers will require advances in scalable manufacturing, early patient stratification, and rationally designed combination regimens to fully realize the clinical potential of CAR-M therapy in PDAC.

### 5.3 Recent Preclinical and Clinical Advances in CAR-NK Cell Therapy for Pancreatic Cancer

#### 5.3.1 Innovative CAR-NK Engineering Strategies

Latest advances in CAR-NK cell therapy for PDAC illustrate a rapidly evolving landscape of innovative engineering strategies aimed at overcoming the profound immunosuppression and stromal complexity of the TME. CD70 has emerged as a compelling dual target, expressed on both malignant cells and CAFs. IL-15-armored CD70-CAR-NK cells exhibit enhanced cytotoxicity, improved persistence, and significant survival benefits in preclinical models through mechanisms involving upregulated CAR expression and autocrine secretion of proinflammatory cytokines [229]. Combination approaches with innate immune agonists, such as STING activator cGAMP, synergize effectively with mesothelin-redirected CAR-NK cells by directly activating NK cell function and increasing tumor cell susceptibility to killing, resulting in superior antitumor responses *in vivo* [230]. Mechanistic insights into CAR-NK biology highlight the transcription factor CREM as a central regulator of activation-induced dysfunction; its deletion enhances antitumor efficacy and promotes epigenetic reprogramming toward a less exhausted phenotype [231]. To counteract TME-driven suppression, Neo-2/15-expressing CAR-NK cells, designed to secrete an IL-2Rβγ superagonist, display improved metabolic fitness and prolonged activity through sustained c-Myc and NRF1 expression, which bolster mitochondrial function and resilience [232]. Additional engineering innovations include CXCR2 overexpression to augment tumor homing and infiltration [233], self-activating CAR-NK cells that release a TGFβ-blocking peptide (P6) to neutralize SMAD-mediated suppression [234], and rational drug combinations with antifibrotic agents such as nintedanib to mitigate CAF-derived IL-6 and restore NK cell cytotoxicity [235]. Beyond conventional targets, CAR-NK cells targeting CD44v6, Robo1, and dual antigen systems (e.g., FRα/DR4) demonstrate potent activity across diverse solid tumors, including PDAC, often leveraging allogeneic “off-the-shelf” platforms suitable for scalable production. Notably, cryopreservable PSCA-targeting CAR-NK cells co-expressing IL-15 have shown robust efficacy in patient-derived models with no significant toxicity, supporting their clinical translatability [236]. Collectively, these multifaceted advances underscore CAR-NK cell therapy as a highly adaptable immunotherapeutic modality capable of targeting both cellular and stromal components of pancreatic cancer.

#### 5.3.2 Emerging Clinical Trial Evidence

Most trials of CAR-NK in pancreatic cancer are ongoing (Table 4). In a phase I clinical trial, researchers developed CAR-NK cells targeting both mucin 1 (MUC1) and PD-1 and administered them to eight patients with advanced cancers, including pancreatic, lung, colon, and ovarian malignancies, that tested positive for both antigens. The treatment was well-tolerated with no severe adverse events reported, and seven patients achieved stable disease throughout the treatment and follow-up period [237]. ROBO1 has also emerged as a potentialtarget for CAR-NK therapy, with three Phase I/II clinical trials currently completed to evaluate its safety and efficacy specifically in pancreatic and other solid tumors; the outcomes of these studies are highly anticipated [238]. Supporting this approach, a recent case report from Shanghai Ruijin Hospital documented a 46-year-old male with unresectable, chemotherapy-refractory PDAC and liver metastases who received repeated infusions of ROBO1-directed CAR-NK cells, including direct intratumoral injections into liver metastases. Within five months, significant reductions in both primary pancreatic and metastatic liver lesions were observed, accompanied by effective disease control. The only notable side effect was transient fever following infusions [239].

**Table 4:** Ongoing clinical trials of CAR-NK therapy in pancreatic cancer

Chimeric antigen receptor	Indication	Phase	Status	Number of patients	Therapeutic approach	Sponsor	Identifier
ROBO1	Pancreatic cancer	I/II	Recruiting	9	ROBO1 specific BiCAR-NK cell	Asclepius technology	NCT03941457
Claudin18.2	Gastric cancer and pancreatic cancer	I	Recruiting	30	Claudin18.2/ Cord blood-derived CAR-NK cell	Zhejiang provincial people’s hospital	NCT06464965
TROP2	Platinum resistant ovarian cancer, mesonephric-like adenocarcinoma, and pancreatic cancer	I/II	Recruiting	51	TROP2-CAR/IL15- transduced CB-NK cell	M.D. Anderson cancer center	NCT05922930
MUC1	Solid tumors (including PDAC)	I/II	Recruiting	10	Anti-MUC1 CAR-pNK cells	PersonGen BioTherapeutics	NCT02839954
NKG2D	Pancreatic cancer	Early Phase I	Recruiting	30	NKG2D CAR-NK	Zhejiang university	NCT06503497
NA	Pancreatic cancer	I	Recruiting	30	CL-NK-001	Changhai hospital	NCT06816823

**Note: **BiCAR-NK: Bispecific Chimeric Antigen Receptor Natural Killer; CAR-pNK: Chimeric Antigen Receptor-precursor Natural Killer; CB-NK: Cord Blood-derived Natural Killer; MUC1: Mucin 1, cell surface associated; NKG2D: Natural Killer Group 2D; PDAC: Pancreatic Ductal Adenocarcinoma; ROBO1: Roundabout Guidance Receptor 1.

#### 5.3.3 Key Challenges in Persistence and Manufacturing

Despite these encouraging results, the clinical efficacy of CAR-NK cells remains limited by their relatively short persistence compared to CAR-T cells [240]. Strategies to extend NK cell durability include brief exposure to cytokines such as IL-12, IL-15, and IL-18, which can induce a memory-like phenotype associated with prolonged activity and enhanced survival [157]. Moreover, several challenges impede the broad application of CAR-NK therapies. Similar to T cells, only a small fraction of infused NK cells successfully infiltrates the tumor microenvironment. The brief *in vivo* half-life of NK cells, approximately 10 days, also necessitates repeated administrations to sustain antitumor effects [241]. From a translational perspective, the selection of optimal cell sources, such as peripheral blood, umbilical cord blood, or pluripotent stem cells, requires careful consideration of scalability, safety (including tumorigenic risks), and standardization of manufacturing protocols to support reproducible and commercially viable allogeneic CAR-NK products [242]. Advances in gene editing and cell culture technologies are expected to address these limitations, thereby accelerating the clinical development and future commercialization of CAR-NK-based immunotherapies for pancreatic and other solid cancers [243].

#### 5.3.4 Translational Gaps in PDAC CAR Therapy

Despite a substantial body of preclinical research exploring CAR-based therapies for PDAC, their translation into clinical trials remains strikingly limited. The scientific community has developed a diverse and sophisticated arsenal of preclinical strategies, centered largely on two paradigms: rational combination therapies and advanced genetic engineering of CAR-T cells. Combination regimens aim to disrupt the immunosuppressive TME by co-administering CAR-T cells with agents such as immune checkpoint inhibitors, conventional chemotherapeutics, or drugs targeting the fibrotic stroma [37803832]. In parallel, the field has engineered increasingly complex “armored” CAR-T cells, which are modified to secrete immunostimulatory cytokines (e.g., IL-12) or express dominant-negative receptors to neutralize inhibitory factors like TGF-β, alongside logic-gated CAR systems engineered for enhanced tumour specificity [107,244].

A significant intellectual gap persists in the systematic evaluation of the limitations inherent to preclinical models, which frequently fail to faithfully recapitulate the intricate heterogeneity and profoundly immunosuppressive nature of the human PDAC TME. Moreover, the field has yet to adequately confront several pivotal path-to-clinic challenges. These encompass the profound heterogeneity of target antigen expression in human PDAC, which can fundamentally undermine mono-targeted approaches, coupled with the substantial practical and financial complexities of manufacturing these next-generation, genetically intricate cellular products in compliance with Good Manufacturing Practice (GMP) standards [110]. The unique pharmacokinetic profiles and the risk of overlapping toxicities when these engineered cells are deployed alongside other systemic therapies in a clinically vulnerable patient population also remain critically underexplored. Absent a more rigorous and candid dissection of these specific obstacles, the successful translation of promising CAR-based therapeutics from bench to bedside for pancreatic cancer will almost certainly remain impeded [245].

## 6 Future Perspective

**Figure 3:**
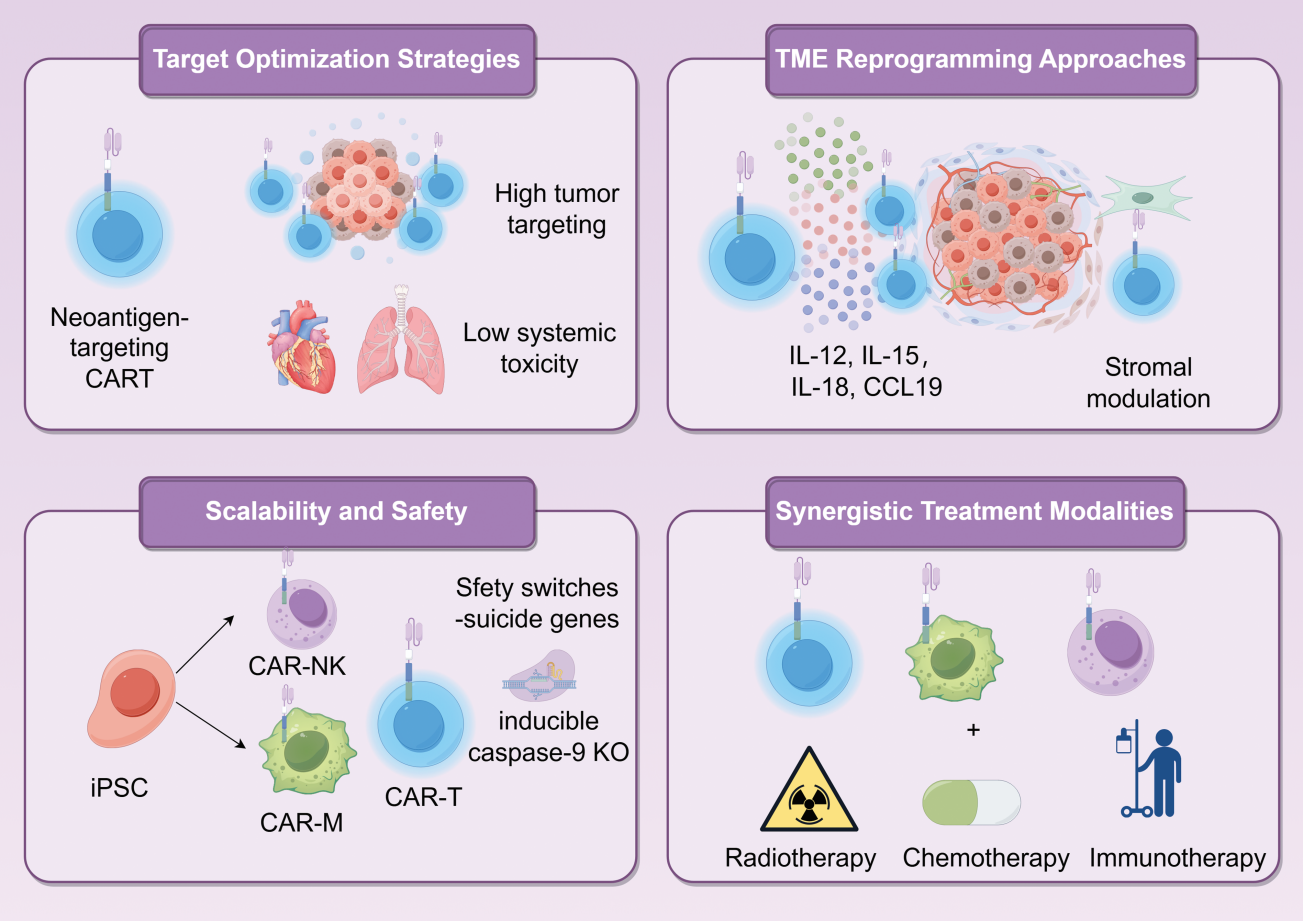
Future directions of next-generation of CAR-immune cell therapy in the treatment of pancreatic cancer. CCL19: C-C Motif Chemokine Ligand 19; KO: Knockout

PDAC remains one of the most challenging malignancies to treat, characterized by a profoundly immunosuppressive TME, dense stromal architecture, and a paucity of effective therapeutic options [246]. While conventional therapies, including chemotherapy, radiation, and even immune checkpoint inhibitors, have provided limited benefit, cellular immunotherapies, particularly those employing CARs, offer a transformative potential [247]. The evolution of CAR-based therapies has expanded beyond T cells to include NK cells and macrophages (CAR-NK, CAR-M), each bringing unique mechanisms for targeting PDAC’s complex biology [123,229] (Fig. 3).

### 6.1 Overcoming Antigen Selection and Tumor Heterogeneity

A primary challenge in CAR therapy for solid tumors like PDAC is the identification of suitable antigens that exhibit high tumor specificity while minimizing on-target, off-tumor toxicity [248]. Traditional targets such as mesothelin, HER2, CEA, and MUC1 have shown promise but are often characterized by heterogeneous expression or shared expression with vital tissues [112]. The emerging paradigm, therefore emphasizes neoantigen discovery and multi-target engineering [249]. Neoantigens, arising from tumor-specific mutations, provide ideal targets due to their absence in normal tissues. Recent bioinformatic and proteomic efforts have identified several candidate neoantigens in PDAC, though their low mutational burden and non-immunogenic relative to other cancers necessitates highly sensitive detection platforms [250]. Additionally, multi-specific CAR designs, such as tandem CARs, dual-targeting CARs, or logic-gated systems, are being developed to enhance specificity and avoid antigen escape [251]. For instance, CAR-T cells targeting both CLDN 18.2 and FAP have demonstrated improved tumor selectivity and reduced toxicity in preclinical models [136]. Similarly, the use of adaptor-based CAR systems, where tumor targeting is controlled by a soluble antibody bridge, allows for tunable activity and improved safety profiles [252]. These strategies not only increase the precision of tumor targeting but also help address the significant inter- and intratumoral heterogeneity characteristic of PDAC.

### 6.2 Reprogramming the Immunosuppressive Tumor Microenvironment

The PDAC TME is a major barrier to CAR immunotherapy, replete with immunosuppressive cells (e.g., regulatory T cells, myeloid-derived suppressor cells), dense fibrosis, and inhibitory cytokines [253]. Next-generation approaches are therefore increasingly focused on reprogramming the TME to support, rather than suppress, immune cell function. Two central strategies have emerged: the use of secretory armored CAR cells and stromal modulation. Armored CARs are engineered to secrete immunomodulatory cytokines such as IL-12, IL-15, IL-18, or CCL19, which can alter the local immune milieu by promoting pro-inflammatory responses, enhancing T or NK cell persistence, and recruiting endogenous immunity [138]. For example, CAR-T cells targeting FAP from CAFs have shown improved infiltration and sustained activity in PDAC models by fostering a favorable cytokine environment [191]. Similarly, CAR-M cells engineered to express pro-inflammatory cytokines or to disrupt fibrotic signaling pathways (e.g., collagen, FAP) can remodel the stromal compartment, enhance phagocytosis, and facilitate deeper penetration of therapeutic cells and drugs [226]. Stromal modulation through targeted depletion of CAFs via FAP-directed CAR-M or CAR-NK cells has also demonstrated significant potential in breaking down physical and biochemical barriers, thereby improving drug delivery and T-cell infiltration.

### 6.3 Enhancing Safety and Mitigating Toxicity

Widespread clinical application of CAR therapies requires solutions to challenges related to manufacturing scalability, treatment-related toxicity, and the use of allogeneic “off-the-shelf” products [254]. Current autologous CAR-T cell therapies are patient-specific, costly, and associated with significant production delays. The development of allogeneic platforms using NK cells or macrophages derived from healthy donors or iPSCs offers a promising alternative [255]. iPSC-derived CAR-NK or CAR-M products can be standardized, mass-produced, and stored as off-the-shelf therapeutics, overcoming key limitations of autologous systems [146]. For example, recent studies have demonstrated the feasibility of generating iPSC-derived CAR-NK cells targeting PSCA or EGFR with robust antitumor activity and a favorable safety profile in PDAC models [143]. To mitigate potential risks such as graft-versus-host disease or uncontrolled immune activation, these platforms often incorporate safety switches, suicide genes (e.g., inducible caspase-9) or surface markers (e.g., truncated EGFR) that allow for precise elimination of engineered cells upon command [256]. The combination of off-the-shelf availability and built-in safety controls significantly enhances the translational potential of next-generation CAR products.

### 6.4 Advancing Scalable and Off-the-Shelf Allogeneic Platforms

The foremost safety challenges associated with CAR cell therapy for pancreatic cancer are manifold. A particularly acute risk is on-target, off-tumor toxicity, a phenomenon arising from the shared expression of tumor-associated antigens on both malignant and healthy tissues. For example, targeting antigens such as HER2 or mesothelin can result in collateral damage to vital organs, including the heart, lungs, and pleura, that physiologically express these proteins at low levels, precipitating severe and potentially life-threatening adverse events [257]. Compounding this issue, patients with advanced pancreatic cancer are frequently immunocompromised as a consequence of their disease burden and prior intensive chemotherapy. This underlying immune deficiency renders them exceptionally susceptible to infectious complications following the lymphodepleting chemotherapy routinely administered prior to CAR-T infusion [258]. Consequently, mitigating this profound iatrogenic immunosuppression in an already vulnerable patient population represents a paramount safety consideration. In response to the challenge of on-target, off-tumor toxicity, innovative engineering strategies are yielding a new generation of “smarter” CAR-T cells. Among these, switchable CAR systems are controlled by administrated bispecific adaptor molecules that function as molecular on/off switches, enabling precise external regulation over T-cell activation. Similarly, logic-gated CARs, designed to require combinatorial antigen recognition, only trigger full activation upon engaging two distinct tumor-associated antigens [259,260]. This Boolean AND-gate logic dramatically enhances tumor specificity and effectively spares healthy cells that express only a single target antigen. To address infection risks, optimized supportive care protocols are being actively refined. These measures include the aggressive implementation of prophylactic antimicrobial regimens, vigilant monitoring for opportunistic pathogens, and the adjunct use of granulocyte colony-stimulating factors (G-CSF) to accelerate hematopoietic recovery and immune reconstitution. For patients at the highest risk, investigation into alternative lymphodepleting regimens with reduced intensity is also underway to better balance efficacy with safety [120].

### 6.5 Developing Rational Combination Therapies

Given the resilience and complexity of PDAC, it is increasingly clear that CAR-immune cell therapies will achieve maximal impact through rational combination regimens. Two particularly promising avenues are radiotherapy priming and small molecule combinations. Radiotherapy has been shown to synergize with CAR therapies by inducing immunogenic cell death, enhancing antigen presentation, and modulating the TME to be more permissive to immune cell infiltration [261]. For instance, proton radiation has been demonstrated to upregulate mesothelin expression in PDAC models and improve the trafficking and efficacy of CAR-T cells [178]. Small molecule inhibitors provide another powerful tool for augmenting CAR therapy. IDO1 inhibitors, JAK/STAT pathway blockers, TGF-β inhibitors, and COX-2 antagonists can counteract key immunosuppressive pathways within the TME [262]. For example, the combination of CAR-T cells with a JAK1/2 inhibitor (ruxolitinib) reversed cytokine-mediated resistance in MUC1-positive PDAC models. Additionally, chemotherapy agents such as gemcitabine or nab-paclitaxel can condition the TME by depleting immunosuppressive cells and enhancing the penetration and persistence of CAR products [219]. The sequential or concurrent application of these modalities with CAR immunotherapy represents a multi-pronged approach essential for overcoming the formidable defenses of pancreatic cancer.

The effective translation of CAR-based cell therapies into PDAC hinges on a stratified patient selection framework, which integrates disease biology, host fitness, and TME features to optimize efficacy and limit risks in heavily pretreated individuals. Stratification first considers disease stage and tumor burden [263]. Patients with low-volume or oligometastatic metastases are often preferred for early-phase trials, whereas those with locally advanced disease may be candidates for neoadjuvant or consolidation regimens [264]. A second essential dimension is target antigen expression and heterogeneity. Robust biomarker profiling, using immunohistochemistry or RNA sequencing of tumor tissue, is required to confirm high, homogeneous antigen levels (e.g., Mesothelin, HER2, CEA, MUC1). Liquid biopsy can supplement this by assessing antigen distribution across metastatic sites. The third stratum involves immune context and TME profiling. Given the immunosuppressive and fibrotic nature of PDAC, stratification may incorporate biomarkers of TME composition, such as cancer-associated fibroblast density or myeloid-derived suppressor cell infiltration, which influence CAR T-cell trafficking and effector function. Prior treatment history is also relevant, as extensive chemotherapy can induce lymphodepletion and compromise T-cell product quality. Finally, clinical eligibility criteria, including good performance status (ECOG 0–1), adequate organ function, and absence of active autoimmunity or infection, are applied. Collectively, this multidimensional approach enables identification of patients most likely to respond, supporting the rational clinical development of CAR-based therapies in PDAC [265].

## 7 Conclusion

In conclusion, the future of CAR-based immune cell therapies in PDAC lies in the intelligent integration of target refinement, microenvironment remodeling, scalable manufacturing, and combination treatments. While significant challenges remain, the rapid pace of innovation in gene editing, cell engineering, and multimodal therapy design offers renewed hope for transforming the prognosis of this devastating disease. Through continued collaboration across disciplines and thoughtful clinical translation, next-generation CAR therapies are poised to become a cornerstone in the treatment of pancreatic cancer (Fig. 3).

## Data Availability

Not applicable.

## Abbreviations

**Term**: **Interpretation**
ACT: Adoptive Cell Therapy
ADCC: Antibody-Dependent Cellular Cytotoxicity
CAFs: Cancer-Associated Fibroblasts
CAR: Chimeric Antigen Receptor
CAR-M: Chimeric Antigen Receptor Macrophage
CAR-NK: Chimeric Antigen Receptor Natural Killer
CAR-T: Chimeric Antigen Receptor T cell
CCL: Chemokine Ligand
CCR: Chemokine Receptor
CD: Cluster of Differentiation
CEA: Carcinoembryonic Antigen
CLDN18.2: Claudin 18.2
COX: Cyclooxygenase
CRS: Cytokine Release Syndrome
CTL: Cytotoxic T Lymphocyte
CXCL: C-X-C Motif Chemokine Ligand
CXCR: C-X-C Motif Chemokine Receptor
DCR: Disease Control Rate
ECM: Extracellular Matrix
EGFR: Epidermal Growth Factor Receptor
EMT: Epithelial-Mesenchymal Transition
FAP: Fibroblast Activation Protein
FasL: Fas Ligand
GM-CSF: Granulocyte-Macrophage Colony-Stimulating Factor
GVHD: Graft-vs.-Host Disease
HER2: Human Epidermal Growth Factor Receptor 2
HIF: Hypoxia-Inducible Factor
ICAM1: Intercellular Adhesion Molecule 1
ICIs: Immune Checkpoint Inhibitors
IDO: Indoleamine 2,3-Dioxygenase
IFN: Interferon
IL: Interleukin
iNOS: Inducible Nitric Oxide Synthase
mOS: Median Overall Survival
MDSCs: Myeloid-Derived Suppressor Cells
MHC: Major Histocompatibility Complex
MMPs: Matrix Metalloproteinases
MSLN: Mesothelin
MUC1: Mucin 1
NK cells: Natural Killer cells
ORR: Objective Response Rate
PBMCs: Peripheral Blood Mononuclear Cells
PD-1: Programmed Cell Death Protein 1
PDAC: Pancreatic Ductal Adenocarcinoma
PD-L1: Programmed Death-Ligand 1
PSCA: Prostate Stem Cell Antigen
PSCs: Pancreatic Stellate Cells
scFv: Single-Chain Variable Fragment
SDF-1: Stromal Cell-Derived Factor 1
SIRPα: Signal-Regulatory Protein α
SMRP: Soluble Mesothelin-Related Peptide
TAA: Tumor-Associated Antigen
TAMs: Tumor-Associated Macrophages
TCR: T Cell Receptor
TGF-β: Transforming Growth Factor Beta
TME: Tumor Microenvironment
TNF-α: Tumor Necrosis Factor Alpha
Tregs: Regulatory T Cells
VEGF: Vascular Endothelial Growth Factor
